# The effectiveness of pediatric obesity prevention policies: a comprehensive systematic review and dose–response meta-analysis of controlled clinical trials

**DOI:** 10.1186/s12967-020-02640-1

**Published:** 2020-12-14

**Authors:** Shahnaz Taghizadeh, Mahdieh Abbasalizad Farhangi

**Affiliations:** 1 Student Research Committee, Department of Community Nutrition, Tabriz University of Medical Sciences, Tabriz, Iran; 2 Drug Applied Research Center, Tabriz University of Medical Sciences, Attar Nishabouri St., POBOX: 14711, 5166614711 Tabriz, Iran

**Keywords:** Childhood obesity, Policy, Prevention, Children, Adolescents

## Abstract

**Background:**

Childhood obesity persists as a serious public health problem. In the current meta-analysis, we summarized the results of controlled trials that evaluated the effect of obesity prevention policies in children and adolescents.

**Methods:**

Three databases (SCOPUS, PubMed and Embase) were searched for studies published before the 6th April 2020, by reported outcome measures of body mass index (BMI) and BMI-Z_score_. Forty-seven studies reported BMI, while 45 studies reported BMI-Z_score_ as final outcome.

**Results:**

The results showed that the obesity-prevention policies had significant effect in reducing BMI (WMD: − 0.127; CI − 0.198, − 0.056; P < 0.001). These changes were not significant for BMI-Z_score_ (WMD: − 0.020; CI − 0.061, 0.021; P = 0.340). In dose–response meta-analysis, a non-linear association was reported between the duration of intervention and BMI (P_nonlinearity_ < 0.001) as well as BMI-Z_score_ (P_nonlinearity_ = 0.023). In subgroup analysis, the more favorite results were observed for 5–10 years old, with combination of physical activity and diet as intervention materials.

**Conclusion:**

In conclusion, the obesity prevention policies in short-term periods of less than 2 years, in rather early age of school with approaches of change in both of diet and physical activity, could be more effective in prevention of childhood obesity.

*Trial registration* PROSPERO registration number: CRD42019138359

## Background

Overweight and obese children persist as a serious health problem and a public challenge of the twenty-first century. Obesity among children and adolescents is a leading cause of health and contributes to cardiovascular disease, cerebrovascular disease, and metabolic diseases [1]. Nearly one in five children and adolescents are overweight or obese [2], and the growing prevalence of obesity in youth has led to an alarming increase of 18.5% in children and adolescents between the ages of 2–19 years [3]. Obese children are at greater risk of obesity in adulthood; a recent study of 200,777 participants showed that 80% of teens with obesity remained obese in adulthood and this continued with a prevalence of 70% past the age of 30 [4]. According to a recent study in the United States comparing the cost–benefit of prevention versus treatment interventions in youth, preventive interventions in the early stages of life were found to be more beneficial than in adulthood, and addressing childhood obesity as early as possible is an effective strategy against obesity in later ages [5]. Although the underlying reasons of genetics and individual behavior for being overweight in adults and young people are almost the same [6], obesity prevention policies in the younger age group are different from those adopted in adulthood. Developing and implementing effective strategies to prevent childhood obesity is difficult at the population level. The National Academy of Sciences recommended that more attention should be paid to providing opportunities to choose healthy foods in society [7]. Obesity prevention is a public health priority around the world. The effectiveness of childhood obesity prevention programs has been shown by previous Cochrane reviews [8]. Some previous systematic reviews have focused on childhood obesity prevention programs that were not at national, governmental or macro-population level policies or that focused on some specific interventional approaches, including changes in physical activity (PA), diet and education [9–13]. Although there is evidence to support the beneficial effects of increased PA and diet as a basic and early strategy at any time and for any age against obesity [14, 15], no summarized study is available to critically evaluate the effectiveness of different policies with different interventional approaches in prevention of childhood obesity considering the role of setting, age, geographical distribution, and intervention type or strategy. Therefore, the aim of the current study was to systematically search controlled trials that evaluated the effectiveness of pediatric obesity prevention policies among children and adolescents and to analyze the effectiveness of these policies on the study outcomes of body mass index (BMI) and BMI-Z_score_ (BMI-Z) measurements while considering a possible dose–response association with preventive tools.

## Methods and materials

The current systematic review and meta-analysis was prepared according to the Preferred Reporting Items for Systematic Reviews and Meta-Analyses (PRISMA) Statement for reporting systematic reviews and meta-analyses [16] (checklist is provided in Additional file 1: Table S1). The study protocol was registered in PROSPERO (identifier: CRD42019138359) and was approved by the Research Undersecretary of the Tabriz University of Medical Sciences as the Ph.D. thesis of SHT (Registration number: IR.TBZMED.REC.1398.840).

### Data sources and search strategy

Searches were conducted using SCOPUS, PubMed and Embase. All articles were considered eligible, if published before April 6, 2020. Additional file 1: Table S2 shows the full search strategy in PubMed. Four concept groups were organized according to the search terms: (a) Population (pediatric, children, or adolescents); (b) Health problem under consideration (obesity, pediatric obesity); (c) Intervention (policy, program, strategy); and (d) Relevant outcomes of interest (BMI, BMI-Z_score)_. The reference lists of all related and available articles were reviewed to reduce the possibility of missing articles. The selection criteria for this review were independently verified by two researchers (SHT, MAF).

### Study selection

Relevant studies conducting a community approach that evaluated policies to prevent obesity in children and adolescents aged 0–18 years were included in the current review. Studies were excluded if they were aimed to treat childhood overweight/obesity), were performed in children with other diseases, or if their full text was not available. Detailed exclusion and inclusion criteria are shown in Table 1.

**Table 1:** Inclusion and exclusion criteria for study selection

	Inclusion criteria	Exclusion criteria
Population	Quantitative studies (e.g., randomized controlled trial, quasi-randomized trials, and cluster randomized trials) Studies evaluating the effect of policies have been done at the macro-population level interventions to prevention childhood obesity Children and adolescent aged 0–18 years Population includes children 0–18 years and outcomes reported separately children 0–18 years	Target group was not children or adolescents (aged > 18 years) Include overweight and obese children Pregnant adolescents Children with disabilities, health conditions (e.g. cystic fibrosis) or behavioural/learning difficulties Studies aimed at treatment childhood obesity Children with eating disorders/disordered eating (e.g. binge eating, bulimia) or other mental health disorders
Intervention	Community-based intervention/program Reports outcomes for children and adolescent Include programs delivered in school (delivered as part of the curriculum or within school hours or after school programmes, changes to school environments/policies (e.g. foods available in the canteen, water fountain installation) Include programs which are primary prevention only Policy changes (e.g. strategies, plans) Environmental changes or interventions—e.g. new parks, water fountain installations Community health service; other community setting (church, sports club, NGO, councils)	Clinical studies (including drugs, single nutrients) Include programs which are secondary prevention Programs which involve clinical treatments (e.g. bariatric surgery) Targets eating disorders/disordered eating (e.g. binge eating, bulimia) or other mental health disorders
Outcomes	Primary or secondary outcomes include BMI or BMI z	Outcomes not reported Primary outcomes diet/healthy eating behaviours or activity-related behaviours such as physical activity Does not report outcomes as BMI or BMI z of interest Does not report outcomes as BMI or BMI z for children and adolescents age 0–18 years Family outcomes only Parent outcomes only
Time	Any duration of intervention	Cross-sectional/observational studies only
Setting	Any country	None
Study type	Intervention studies (e.g. RCT, non-randomised experimental); full scale and pilot implementation studies	Intervention pre-post studies without control group, small scale Intervention not in the macro-population level
Publication year Any	Any	Any
Other	Article/abstract in any language	Abstract only Review article Editorials Conference abstracts Letters Commentaries Study protocols

### Quality assessment and data extraction

Study quality was assessed using the Effective Public Health Practice Project Quality Assessment Tool for Quantitative Studies, a useful tool for quality assessment of randomized and non-randomized intervention trials [17, 18]. This tool is comprised of six components that include selection bias, study design, confounders, blinding, data collection methods (validity/reliability), and withdrawals and dropouts. The overall quality rating and the components are scored as strong, moderate and weak according to the tool’s instructions. Individual component quality rankings are shown in Additional file 1: Table S3. General study characteristics (author, year of publication, country, sample size, number of intervention and control, type of study (randomized or non-randomized), duration of intervention, follow-up from baseline, follow-up from end of intervention, participant characteristics, outcomes (BMI, BMI-Z_score_), and policy characteristics were extracted for included studies. Effect size was defined as changes in BMI and BMI-Z_score_ compared with control group. Two researchers (SHT, MAF) independently extracted the data from all studies.

### Statistical analysis

The data were analyzed using STATA version 15 (STATA Corp, College Station, TX, USA), and *p-*values of less than 0.05 were considered statistically significant.

#### Two-class meta-analysis of continuous variable

The studies that reported BMI and BMI-Z_score_ as primary or secondary outcomes in intervention and control groups were included for two-class meta-analysis synthesis. The means and standard deviations (SD) of variables were used to compute standardized mean differences as effect size computed by pooled estimate of weighted mean difference (WMD) at a 95% confidence interval (CI). Subgroup analyses were conducted to explore sources of heterogeneity. Due to high heterogeneity values (i.e., above 50%), the random effects model was used. Between-study heterogeneity was identified using Cochran's Q and I-squared tests as follows: I^2^ < 25%, no heterogeneity; I^2^ 25% to 50%, moderate heterogeneity; I^2^ > 50%, large heterogeneity [19]. Studies that reported separate results for both sexes, in different age categories, or at different time periods of follow-up were included as individual studies. Publication bias was examined using Begg’s funnel plots, followed by Egger's regression asymmetry test and Begg's rank correlation for formal statistical assessment of funnel plot asymmetry. For missing SDs, the method described by Walter and Yao was used to calculate SD [20]. Studies were excluded from the analysis if they (a) were not controlled trials or (b) did not report sufficient data of outcome variables.

#### Dose–response meta-analysis of continuous variables

For dose–response meta-analysis of variables, variables of duration of intervention and PA time and training sessions (as education time) were included. The mean difference of variables in each study was also identified. A dose–response meta-analysis of BMI and BMI-Z_score_ was performed using fractional polynomial modeling [21] to explore nonlinear potential effects of duration of intervention (year), PA and education time and study-specific parameters.

## Results

### Literature search and study characteristics

**Fig. 1:**
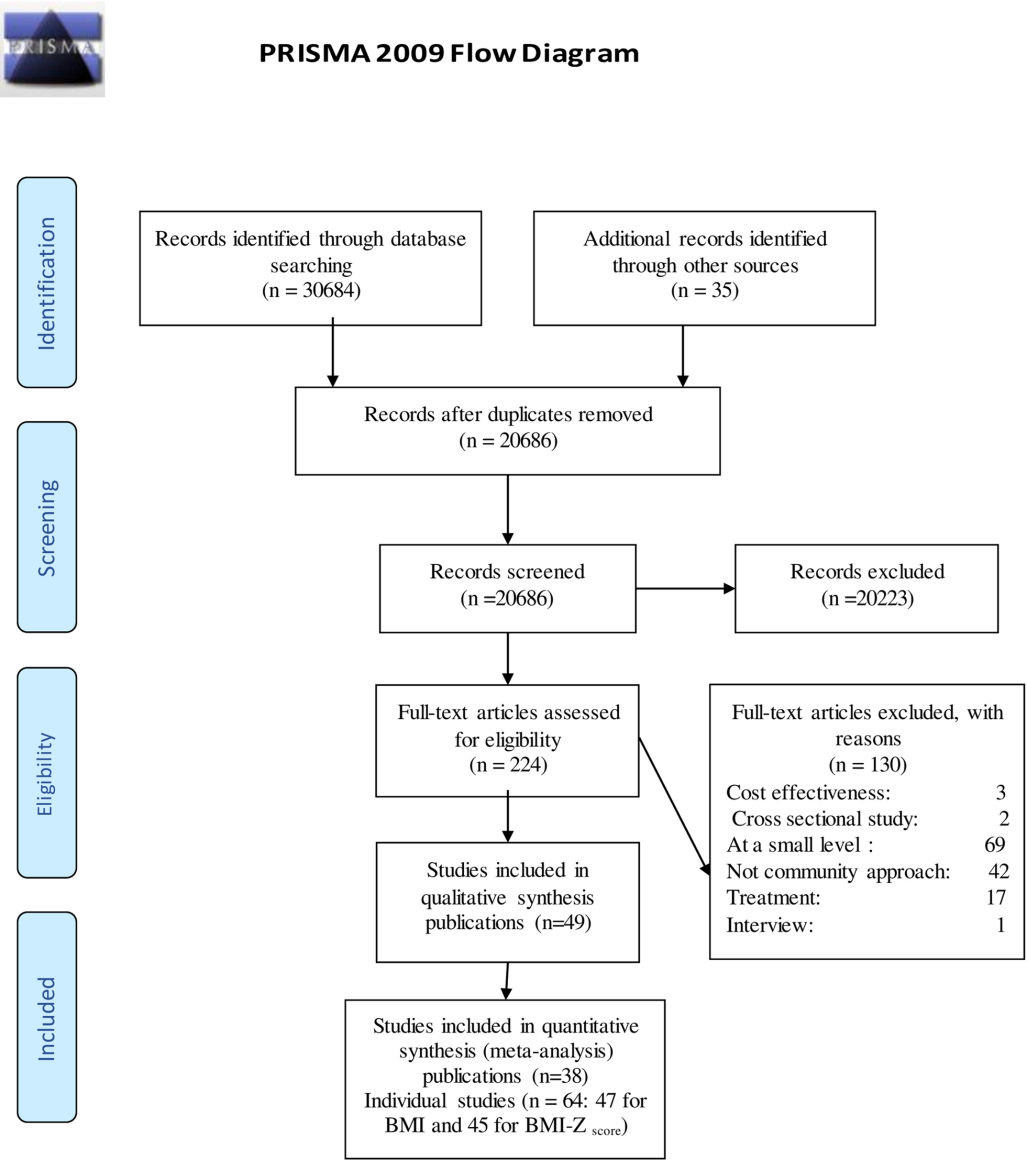
Flow chart of study selection

A search of electronic data bases retrieved 30,719 records. After removing duplicates, 20,686 items were screened by title/abstract (Fig. 1) and selected according the criteria identified above. The remaining 224 full text articles were screened and 49 publications were selected in a qualitative synthesis; finally, 38 publications were included in a quantitative synthesis, which contained outcomes for 64 individual studies as described above.

Grey literature searches identified no published results for policies in scope. Study, participant, and program characteristics of the quantitative synthesis (meta-analysis) are presented in Table 2 with additional information including the full name of the studies shown in Additional file 1: Table S4. Studies were performed in various settings of school (n = 16) [22–37], community and school (n = 10) [38–47], school and home (n = 1) [48], community, school, and home (n = 2) [49, 50], community, school, home, and primary care clinic (n = 5) [51–55], community and home (n = 2) [56, 57], primary care clinic (n = 1) [58], and cyberspace/online (n = 1) [59]. In all, 64 individual studies were obtained from 38 publications included in the quantitative synthesis. Twelve studies were performed as combinations of different follow-up times, age groups, genders, or different durations or populations; therefore each was included as two [23–25, 31, 35, 36, 42, 44, 48, 50, 54–56, 59], three [41, 49, 52], or four individual studies [30, 51]. The rationale for extracting several studies from these publications and additional information about the policies are shown in Table 2 and Additional file 1: Table S5). Characteristics of studies that were not included in the meta-analysis with the exclusion reasons are shown in Additional file 1: Table S6.

**Table 2:** The general characteristics of the studies included in the meta-analysis of the association between childhood obesity prevention policies and Body mass index (BMI) and BMI-Z_score_

Setting (N of studies)	First Author/Year of publish/(reference)	Main focus	Intervention^a^	Study type^b^	Country/name of program	Increase of PA^c^	Session^d^	Total sample (IN, CN)	Duration (year)	Range of age	Follow-up (year)^e^	Frequency of intervention	Target group	Quality score^f^	^g^↓ in BMI	^g^↓ in BMI-Z
School (n = 25)	Wang/2018 [22]	PA	2	1	China/YOG-Obesity study	NR	NR	9858 (5275, 4583)	1	9–12	0^h^	W	Children	1	✔	✔
	Leme/2018 [23] (two individual study due to follow up)	Diet + PA	0	1	Brazil/H3G-Brazil	NR	60	253 (412, 111)	0.5	14–18	0, 0.5	M	Children and parents	3	–	–
	Lubans/2016 [24] (two individual study due to follow up)	Diet + PA	2	1	Australia/ATLAS	26	3	361 (181, 180)	0.66	12–14	0, 0.84	W	Children	1	–	–
	Hollis/2016 [25] (two individual study due to follow up)	Diet + PA	2	1	Australia/PA4E1	70,156	288	1150 (645, 505)	1, 2	10–11	0	W	Children	2	✔	✔
	Smith/2014 [26]	PA	2	1	Australia/ATLAS	18	1.5	361 (181, 180)	0.41	12–14	0.25	W	Children	2	–	^j^
	Lubans/2012 [27]	Diet + PA	2	1	Australia/NEAT Girl	91.5	4.6	357 (178, 179)	1	12–14	0	W	Children	1	–	–
	Millar/2011 [28]	Diet + PA	2	1	Australia/IYM	NR	NR	2054 (1276, 778)	1	12–18	1.3	D	Children	1	✔	✔
	Llargues/2011 [29]	Diet + PA	2	1	US/AVall	NR	288	509 (272, 237)	2	5–6	0	W	Children	1	–	^j^
	Salcedo Aguilar/2010 [30] (four individual study due to sex and duration)	PA	2	1	New Zealand/MOVI	234,468	126	921 (375, 546)	1, 1.66	9–10	0	W	Children	1	–	^j^
	Neumark-Sztainer/2010 [31] (two individual study due to follow up)	Diet + PA	2	1	US/New Moves	32	27.33	356 (182, 147)	0.33	14–189	0, 0.41	W	Children	1	–	^j^
	Group/2010 [32]	Diet + PA	2	1	US/school-based program on risk factors for DM	NR	NR	4603 (2307, 2296)	2	11–12	0	NR	Children	3	^i^	✔
	Dzewaltowski/2010 [33]	Diet + PA	2	1	US/HOP’N	215	240	273 (148, 125)	2	9–10	0	D	Children	3	–	–
	Donnelly/2009 [34]	PA	2	1	US/PAAC	234	NR	1527 (814, 713)	3	6–9	0	W	Children	1	–	^j^
	Taylor/2008 [35] (two individual study due to follow up)	Diet + PA	2	2	New Zealand/APPLE	NR	NR	727 (381, 346)	1	6–11	0, 1.8	D	Children	3	NR^k^	✔
	Martínez Vizcaíno /2008 [36] (two individual study due to sex)	PA	2	1	Spain/Movi	108	NR	1119 (513, 579)	0.5	9–10	0	W	Children	2	–	^j^
	Foster/2008 [37]	Diet + PA	2	1	US/SNPI	NR	180	1349 (749, 600)	2	9–12	0	W	Children	2	^i^	–
Community, school (n = 14)	Bell/2019 [38]	Diet + PA	3	1	Australia/OPAL	NR	NR	2353 (1208, 1145)	5	0–18	0	D	Children and parents	3	–	^j^
	Santiago Felipe/2018 [39]	Diet + PA	3	1	Spain/TCHP	NR	30	2086 (974, 112)	1.25	8–10	0	W	Children and parents	3		–
	Novotny/2018 [40]	Diet + PA	3	1	US/Children’s Healthy Living Program	NR	NR	1882 (952, 930)	2	2–8	0	NR	Children	2	^i^	–
	Adab/2018 [41] (three individual study due to follow up)	Diet + PA	1	1	UK/WAVES	45	21	1392 (660, 732)	1	6–7	0.25, 1.5, 2.25	D	Children	3	^i^	–
	Sadeghi/2017 [42] (two individual study due to sex)	Diet + PA	0	1	US/NSFS	10.4	NR	422 (271, 151)	3	3–8	1	W	Children	1	^i^	–
	Swinburn/2014 [43]	Diet + PA	3	1	Australia/BAEW	NR	NR	1674 (877, 797)	3	10–12	0	D	Children	1	–	–
	Pettman/2014 [44] (two individual study due to age groups)	Diet + PA	3	1	Australia/ewba	NR	NR	2631 (1300, 1331)	3	4–5, 10–12	0	NR	Children	2	–	–
	Kremer/2011 [45]	Diet + PA	3	1	Fiji/HYHC	NR	NR	2968 (879, 2069)	1.75	13–18	0	NR	Children	2	–	–
	Fotu/2011 [46]	Diet + PA	3	1	Tonga/MYP	NR	NR	1712 (815, 897)	2.4	11–19	0	NR	Children and parents	3	–	–
	Sanigorski/2008 [47]	Diet + PA	3	2	Australia/BAEW	NR	NR	3688 (1001, 2687)	3	4–12	0	D	Children	2	–	✔
School, home (n = 2)	Romon/2009 {48] (two individual study due to sex)	Diet + PA	2	2	France/FLVS	NR	NR	1502 (804, 698)	12	5–12	0	NR	Children	1	✔	^j^
Community, school, home (n = 5)	Crespo/2012 [49] (three individual study due to follow up)	Diet + PA	3	1	US/APN	NR	22	392 (165, 227)	1	5–7	0, 1, 2	NR	Children	3	^i^	–
	Gentile/2009 [50] (two individual study due to duration)	Diet + PA	3	1	US/ Switch& what you Do, View, and Chew	NR	NR	1323 (670, 653)	6,12	6–11	0	NR	Children	2	–	^j^
Community, school, home, primary care clinic (n = 12)	Economos CD /2007 [51] (four individual study due to community and sex)	Diet + PA	3	2	US/SUS	40	16	1178 (385, 793)	0.66	6–8	0	W	Children and parents and teachers and policy makers	2	^i^	✔
	Wong/2016 [52] (three individual study due to follow up)	Diet + PA	2	1	US/Healthy Kids Houston	NR	NR	877 (524, 353)	0.125, 0.25, 0.375	9–12	0, 0.125, 0.25	W	Children	1	–	–
	Johnson/2012 [5])	Diet + PA	3	1	Australia/BAEW	NR	NR	2905 (1726, 1183)	3	4–12	0	NR	Children	3	NR^k^	✔
	de Silva-Sanigorski /2010 [54] (two individual study due to age groups)	Diet + PA	3	1	Australia/Romp and Chomp	NR	NR	35,157 (2778, 32,379)	3	0–5	3	D	Children	1	✔ (only in 2 years old)	✔ (only in 2 years old)
	Taylor/2007 [55] (two individual study due to duration)	Diet + PA	3	2	New Zealand/APPLE	NR	NR	470 (251, 219)	1,2	5–12	0	NR	Children	1	–	✔
Community, home (n = 3)	de Henauw/2015 [56] (two individual study due to sex)	Diet + PA	3	2	8 European countries/IDEFICS	NR	NR	16,228 (4882, 7746)	0.58	2–9.9	1.42	NR	Children and parents	1	^i^	✔ (in girls)
	Elder /2014 [57]	Diet + PA	3	1	US/MOVE	NR	36.6	541 (271, 270)	2	10–14	0	W	Parent	3	–	–
Primary care clinic (n = 1)	Eno Persson/2018 [58]	Diet + PA	0	2	Sweden/PRIMROSE	NR	NR	1030 (431, 599)	3.25	0.75–5	1	NR	Parent	3	–	^j^
Cyberspace (n = 2)	Hammersley/2019 [59] (two individual study due to follow up)	Diet + PA	0	1	Australia/Time2bHealthy	NR	NR	86 (42, 44)	0.5	2–5	0.25, 0.5	M	Parent	3	–	^j^

*D* daily, *W* weekly, *M* monthly, *NR* not reported

^a^ 0: Only education, 1: education as curricula, 2: education + change in school environment (such as increased PA or changes in school diet), 3: involvement other community sections)

^b^ 1: Randomized controlled-trials(RCT), 2: Non-randomized controlled-trials

^c^ Total hours increase of PA in the duration of intervention

^d^ Educational session was held in the duration of intervention

^e^ Follow-up from end of intervention

^f^ 1: weak, 2: moderate, 3: strong, Component scores for quality rating are included in Additional file 1: Table S4

^g^ Tickets (**✔**) show a significant decrease (P < 0.05) in the body mass index (BMI) or BMI Z_score_ (BMI-Z)

^h^ Follow up 0 means: Immediately After the End of the Intervention

^i^ BMI was not as outcomes

^j^ BMI-Z was not as outcomes

^k^ BMI was among the outcomes, but no significant changes were reported

Approximately 35% of programs were carried out in the United States (n = 13) [29, 31–34, 37, 40, 42, 49–52, 57], and 31% (n = 12) studies in Australia [24–28, 38, 43, 44, 47, 53, 54, 59]. Other studies took place in China (n = 1) [22], Brazil (n = 1) [23], New Zealand (n = 3) [30, 35, 55], Spain (n = 2) [36, 39], the United Kingdom (n = 1) [41], Fiji (n = 1) [45], Tonga (n = 1) [46], France (n = 1) [48], Sweden (n = 1) [58], and one study which was conducted in eight European countries (Belgium, Cyprus, Estonia, Germany, Hungary, Italy, Spain and Sweden) [56].

Thirty studies reported BMI [22–31, 33–36, 38, 39, 43–48, 50, 52–55, 57–59] and 27 studies reported BMI-Z_score_ [22–25, 27, 28, 32, 33, 35, 37, 39–47, 49, 51–57]. The total number of participants in the systematic reviews was 200,255; 178,017 participants were included in the meta-analysis, ranging from 86 [59] to 35,157 [54], with an average sample size of 2849. Nine studies were carried out among girls, [23, 27, 30, 31, 36, 42, 48, 51, 56], eight studies among boys [24, 26, 30, 36, 42, 48, 51, 56] and 21 studies were performed with both genders. The majority of policies (n = 33) examined combined diet and PA interventions, with five studies that consisted of only PA [22, 26, 30, 34, 36] and no study focused only on diet. The majority of studies (n = 31) were conducted as randomized controlled trials (81.5%), and seven [35, 47, 48, 51, 55, 56, 58] were non-randomized controlled trials (18.4%). BMI or BMI-Z_score_ as outcomes were reported at the end of the intervention in 31 studies [22–27, 29–40, 43–53, 55, 57], and 14 programs had follow-up periods after the end of the intervention [23, 24, 26, 28, 31, 35, 41, 42, 49, 52, 54, 56, 58, 59]. The length of follow-up ranged from 6 weeks [52] to 3 years [54].

### Dose–response meta-analysis of the association between education time, PA, duration of intervention and BMI or BMI-Z_score_

**Fig. 2:**
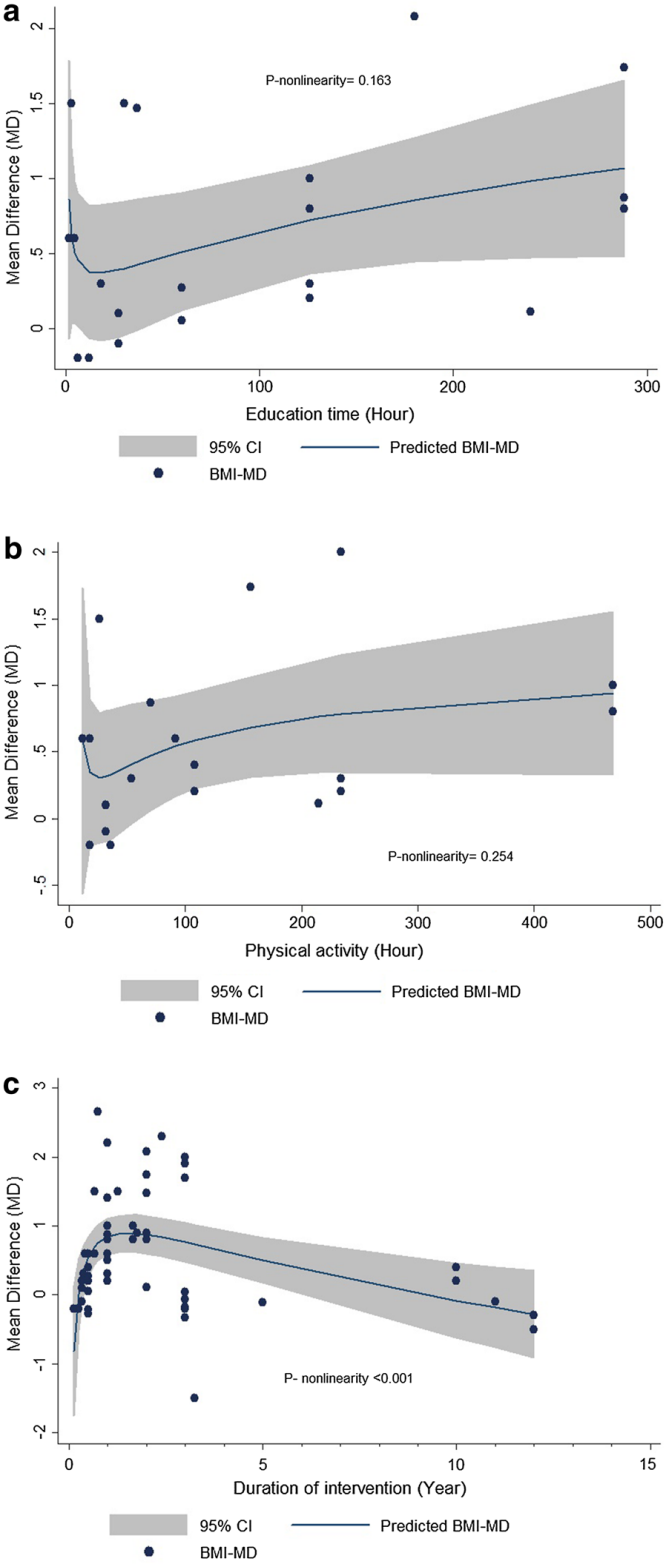
Dose–response association between duration of intervention, PA, education time and body mass index (BMI). Linear relation (solid line) and 95% confidence interval (CI) (gray area) of mean difference in BMI. This figure indicates the association between mean difference of BMI and **a** education time, **b** PA, **c** duration of intervention

**Fig. 3:**
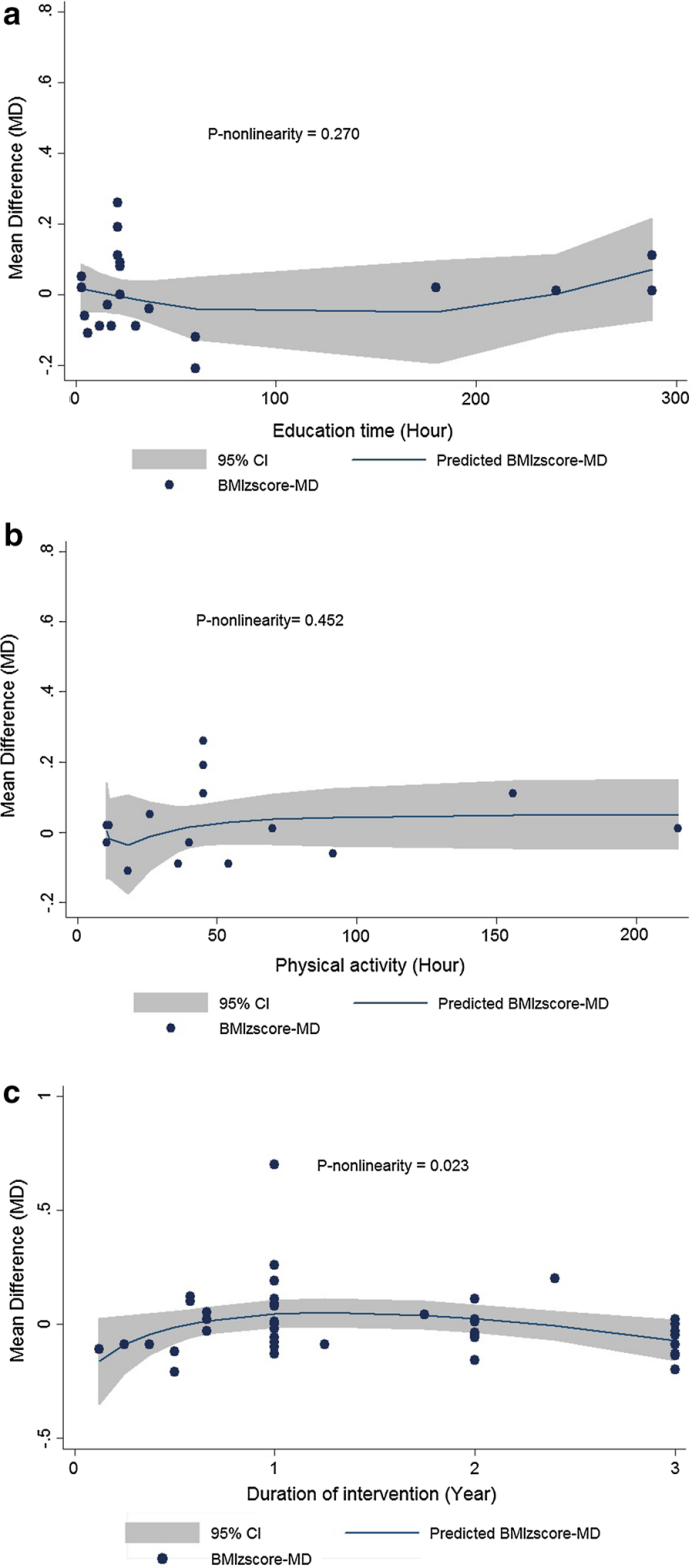
Dose–response association between duration of intervention, PA, education time and BMI-Z_score_. Linear relation (solid line) and 95% confidence interval (CI) (gray area) of mean difference in BMI-Z. This figure indicates the association between mean difference of BMI-Z and **a** education time, **b** PA, **c** duration of intervention

The non-linear dose–response association between the study outcomes of BMI or BMI-Z_score_ and education time, PA, and duration of intervention was performed using fractional polynomial (FP) modelling. Thirteen studies were assessed for a dose–response association between BMI and education time [23–27, 29–31, 33, 37, 39, 52, 57], and 12 studies for BMI-Z_score_ and education time [23–25, 27, 33, 37, 39, 41, 49, 51, 52, 57] (Figs. 2a, 3a). There was no evidence for nonlinear association between BMI (P- for nonlinearity = 0.163) or BMI-Z_score_ (P- for nonlinearity = 0.270) with education time. Ten studies were assessed for a dose–response association between BMI and PA [24–27, 30, 31, 33, 34, 36, 52] and 8 studies for BMI-Z_score_ [24, 25, 27, 33, 41, 42, 51, 52] (Figs. 2b, 3b). No evidence of nonlinearity association was observed between BMI (P- for nonlinearity = 0.254) or BMI-Z_score_ (P- for nonlinearity = 0.452) and PA. All 30 studies of BMI and 27 studies of BMI-Z_score_ were included for calculating the dose–response association between changes in BMI or BMI-Z_score_ with duration of intervention, respectively (Figs. 2c, 3c). There was evidence of a nonlinear association between the duration of intervention and BMI (P- for nonlinearity < 0.001) as well as BMI-Z_score_ (P- for nonlinearity = 0.023).

Details of the dose–response association between duration of intervention, PA, education time and BMI and BMI-Z_score_ are shown in Table 3.

**Table 3:** Details of non-linear association between BMI and BMI-Z_score_ with study specific parameters

BMI^a^_Mean difference_	Coefficient	Standard error	t	P >|t|	95% Conf. Interval
Education time					
Dose_1	0.3085	0.1785	1.73	0.100	− 0.0652, 0.6822
Dose_2	0.0855	0.0588	1.45	0.163	− 0.0376, 0.2087
_cons	0.6306	0.1707	3.69	0.002	0.2733, 0.9879
Physical activity hour^b^					
Dose_1	0.2787	0.2893	0.96	0.350	− 0.3347, 0.8921
Dose_2	− 1.0968	0.9264	− 1.18	0.254	− 3.0608, 0.8671
_cons	0.6516	0.1710	3.81	0.002	0.2889, 1.0142
Duration of intervention					
Dose_1	− 1.0312	0.2833	− 3.64	0.001	− 1.6001, − 0.4624
Dose_2	− 0.2733	0.0730	− 3.74	*< 0.001*	− 0.4200, − 0.1267
_cons	0.8181	0.1377	5.94	< 0.001	0.5415, 1.0946
BMI-Z_score Mean difference_					
Education time					
Dose_1	− 0.1331	0.1319	− 1.01	0.325	− 0.4075, 0.1413
Dose_2	0.0523	0.0462	1.13	0.270	− 0.0437, 0.1484
_cons	− 0.0395	0.0416	− 0.95	0.353	− 0.1261, 0.0470
Physical activity hour^b^					
Dose_1	− 0.0103	0.0128	− 0.80	0.435	− 0.0377, 0.0171
Dose_2	− 0.0043	0.0055	− 0.77	0.452	− 0.0162, 0.0075
_cons	0.0291	0.0287	1.01	0.327	− 0.0321, 0.0903
Duration of intervention					
Dose_1	0.7926	0.3537	2.24	0.030	0.0788, 1.5064
Dose_2	− 0.3482	0.1474	− 2.36	*0.023*	− 0.6458, − 0.0505
_cons	0.0487	0.0307	1.58	0.121	− 0.0134, 0.1108

The significant P-values of Dose_2 are presented as italic numbers

^a^Body mass index

^b^This refers to the hours of physical activity other than the normal physical activity that takes place in the school's physical activity course

### Two-class meta-analysis of the comparison of effectiveness of childhood obesity prevention policies on BMI and BMI-Z_score_

**Fig. 4:**
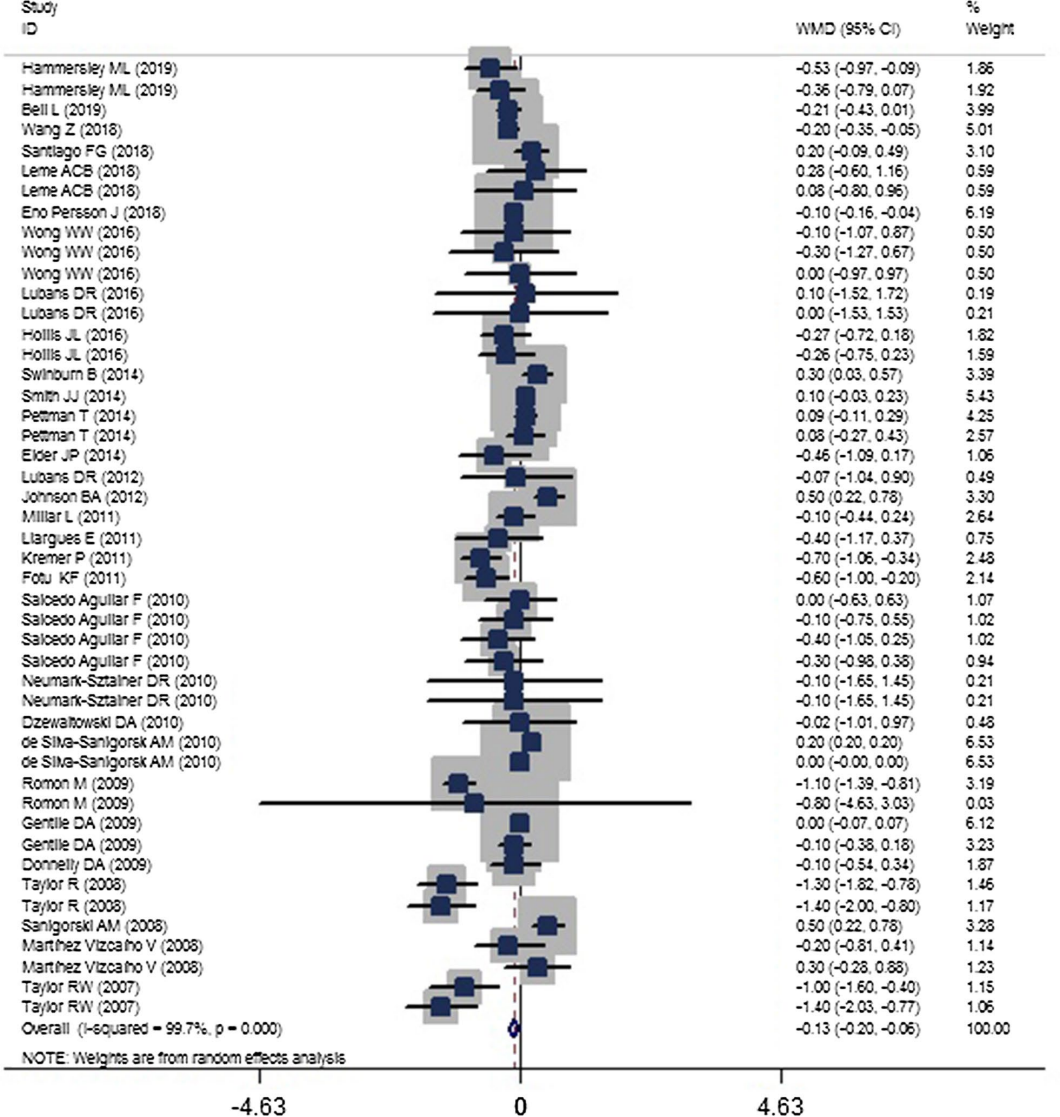
The forest plot showing the weighted mean difference (WMD) of the effect of childhood obesity prevention policies on body mass index (BMI) [weighted mean difference (WMD): − 0.127; confidence interval (CI) − 0.198, − 0.056; P < 0.001]

**Fig. 5:**
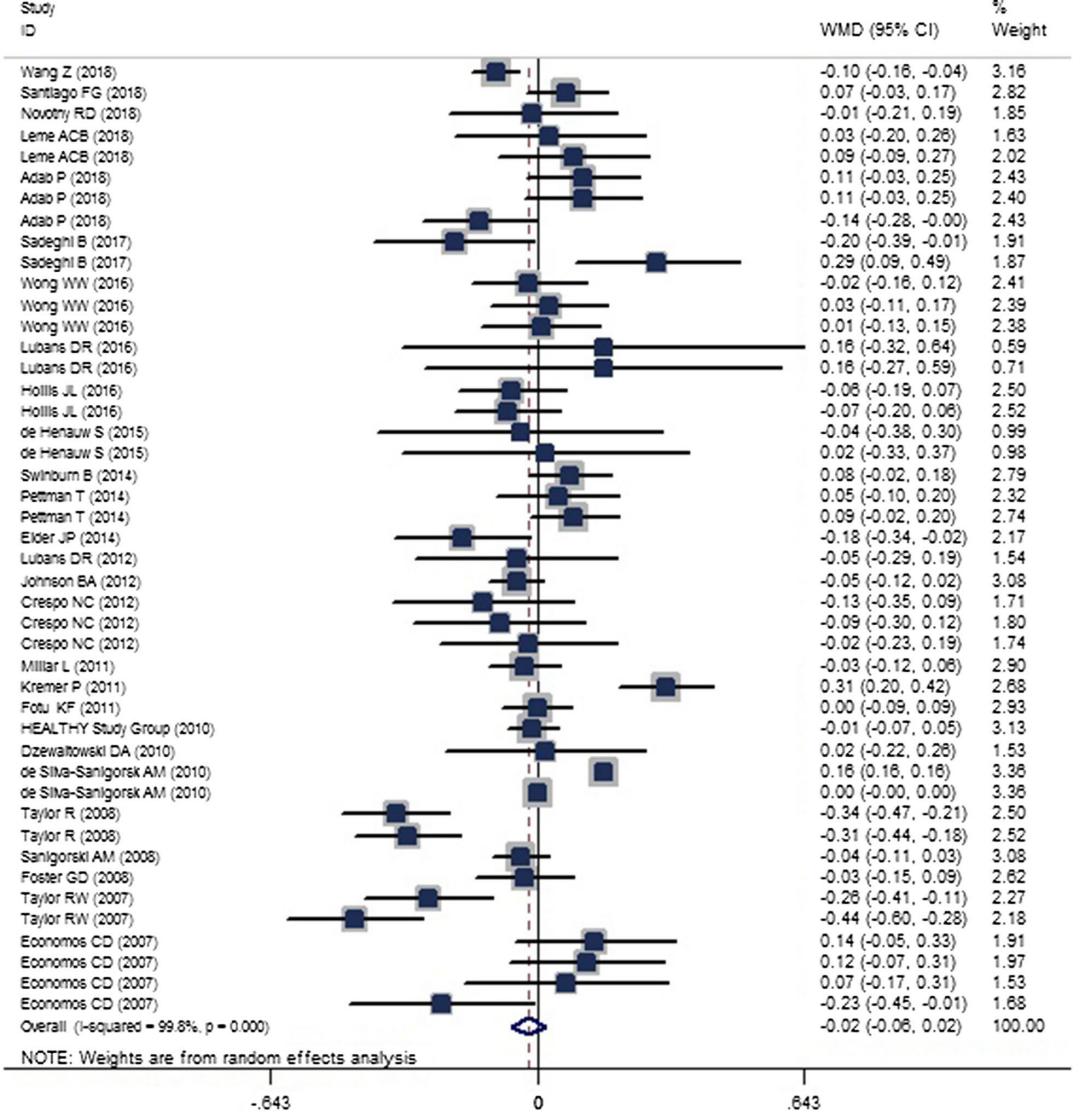
The forest plot showing the weighted mean difference (WMD) of the effect of childhood obesity prevention policies on body mass index Z score (BMI-Z_score_) [weighted mean difference (WMD): − 0.020; confidence interval (CI) − 0.061, − 0.021; P = 0.340]

A total of 38 publications [22–59] were included in the two-class meta-analysis of the effects of obesity prevention policies on BMI (Fig. 4) and BMI-Z_score_ (Fig. 5).

The results showed that obesity-prevention policies had a significant effect in reducing BMI (WMD: − 0.127; CI − 0.198, − 0.056; *P* < 0.001; I^2^ = 99.7%; P-heterogeneity < 0.001) and a non-significant reduction in BMI-Z_score_ (WMD, − 0.020; CI − 0.061, − 0.021; *P* = 0.340; I^2^ = 99.8). A subgrouping meta-analysis (shown in Tables 4 and 5) and a meta-regression (Table 6) were also performed to assess the source of heterogeneity for the included studies. According to the subgroup meta-analysis, school-based policies in children aged 5–10 years, in relatively short period of time (less or equal to 2 years), with approaches to practical changes in diet and PA (i.e., not consisting of education only) and the policies that were performed in combination with both genders seemed to be more effective in reducing BMI and BMI-Z_score_ with more favorable changes. Subgrouping also revealed that the heterogeneity level for BMI was reduced in subgrouping according to target group (e.g., for the parent group it was reduced from 99.7 to 49.8%), type of intervention (e.g., for only education it was reduced from 99.7 to 30.9%), study focus (e.g., for PA it was reduced from 99.7 to 35.7%), and frequency of intervention (e.g., for monthly it was reduced from 99.7 to 13.4%). In examining setting, the setting of community, school, and home and school, home and cyberspace and continent as US, the frequency of intervention as weekly, baseline BMI as a range of 22–25 and ≥ 25 kg/m^2^, and gender as male, heterogeneity disappeared. For BMI-Z_score_, the target group, the continent, the gender, and the setting were the primary sources of heterogeneity.

**Table 4:** Results of subgroup analysis for the effects of childhood obesity policies on childhood BMI

Group	No. of trial	WMD (95% CI)			P	P _heterogeneity_	I^2^, %
Total	47	− 0.127	− 0.198	− 0.056	< *0.001*	< 0.001	99.7
Setting							
School	23	− 0.225	− 0.398	− 0.053	*0.01*	< 0.001	60.7
Community, school, home	2	− 0.006	− 0.075	0.063	0.864	0.5	0
Community, school	8	− 0.027	− 0.285	0.231	0.839	< 0.001	85.1
School–home	2	− 1.098	− 1.383	− 0.814	0.155	0.878	0
Community, home	1	− 0.46	− 1.094	0.174	< *0.001*		
Community	8	0.007	− 0.151	0.166	0.93	< 0.001	100
Primary care clinic	1	− 0.1	− 0.165	− 0.035	*0.002*		
Cyberspace	2	− 0.443	− 0.751	− 0.135	*0.005*	0.589	0
Target group							
Children	38	− 0.109	− 0.19	− 0.029	*0.008*	< 0.001	99.8
Parent	4	− 0.276	− 0.522	− 0.031	*0.028*	0.113	49.8
Children and parents	5	− 0.112	− 0.435	0.211	0.497	0.019	66
Continent							
USA	13	− 0.016	− 0.083	0.05	0.632	0.976	0
Europe	6	− 0.208	− 0.656	0.24	0.364	< 0.001	90.5
Oceania	27	− 0.109	− 0.198	− 0.02	*0.017*	< 0.001	99.8
Asia	1	− 0.2	− 0.353	− 0.047	*0.01*		
Intervention content^a^							
Education	5	− 0.185	− 0.391	0.022	0.081	0.216	30.9
Education as curricula		–	–		–	–	–
Education + change in school environment (such as increased PA or changes in school diet)	26	− 0.302	− 0.501	− 0.102	*0.003*	< 0.001	74.6
Other community sections	16	− 0.009	− 0.105	0.088	0.862	< 0.001	99.9
Study focus							
Diet + PA	38	− 0.14	− 0.219	− 0.061	*0.001*	< 0.001	99.8
PA	9	− 0.065	− 0.216	0.086	0.397	0.132	35.7
Age-category							
< 5 years old	6	− 0.022	− 0.158	0.114	0.751	< 0.001	100
5–10 years old	22	− 0.3	− 0.52	− 0.08	*0.008*	< 0.001	87.5
≥ 10 years old	19	− 0.133	− 0.28	0.014	0.077	0.009	48.7
By sample size							
≤ 1000	28	− 0.388	− 0.632	− 0.143	0.002	< 0.001	78
1000–2000	10	− 0.044	− 0.146	0.057	0.393	0.008	59.4
≥ 2000	9	0.037	− 0.08	0.154	0.531	< 0.001	100
Frequency of intervention							
Daily	9	− 0.023	− 0.154	0.108	0.73	< 0.001	100
Weekly	22	− 0.042	− 0.121	0.038	0.303	0.541	0
Monthly	4	− 0.302	− 0.61	0.005	0.054	0.326	13.4
NR^b^	12	− 0.127	− 0.198	− 0.056	*0.002*	< 0.001	90.7
Duration of intervention (years)							
≤ 1	25	− 0.243	− 0.388	− 0.098	*0.001*	< 0.001	67.5
1–2	9	− 0.38	− 0.725	− 0.036	*0.03*	< 0.001	72.1
> 2	13	− 0.006	− 0.109	0.098	0.917	< 0.001	99.9
Follow up from baseline (years)							
≤ 1	21	− 0.114	− 0.223	− 0.006	*0.039*	0.047	36.8
1–2	10	− 0.366	− 0.697	− 0.035	*0.03*	< 0.001	99.9
> 2	16	− 0.077	− 0.176	0.021	0.122	0.001	68.7
Sex							
Boys and girls	31	− 0.111	− 0.19	− 0.033	*0.005*	< 0.001	99.8
Girls	9	− 0.209	− 0.684	0.265	0.387	< 0.001	73.5
Boys	7	0.077	− 0.04	0.195	0.197	0.96	0
Baseline BMI^c^							
≤ 18	18	− 0.142	− 0.244	− 0.041	*0.006*	< 0.001	99.9
18–22	20	− 0.09	− 0.186	0.006	0.065	0.017	44.4
22–25	7	− 0.291	− 0.568	− 0.015	*0.039*	0.508	0
≥ 25	2	− 0.1	− 1.193	0.993	0.858	1	0
Quality rating							
Strong	13	− 0.294	− 0.531	− 0.056	0.015	< 0.001	84
Moderate	23	− 0.17	− 0.284	− 0.056	0.899	< 0.001	71.1
Weak	11	− 0.009	− 0.149	0.131	0.003	< 0.001	99.9

The twelve studies was included as two individual studies [2–4, 10, 14, 15, 23, 27, 29, 33, 34, 38], one study as three individual studies [31], and one study as four individual studies [9]; the significant P-values are presented as italic numbers

*WMD* weighted mean difference, *PA* physical activity

^a^Education, is various training that can be different based on policies, but education as curricula is a unit of instruction in schools that is done as course regularly during the school year

^b^Not reported

^c^Body mass index

**Table 5:** Results of subgroup analyses for the effects of childhood obesity policies on childhood BMI-Z_score_

Group	No. of trial	WMD (95% CI)			P	P_heterogeneity_	I^2^, %
Total	45	− 0.02	− 0.061	0.021	0.34	< 0.001	99.8
Setting							
School	14	− 0.073	− 0.137	− 0.01	*0.024*	< 0.001	69.4
Community, school, home	7	− 0.08	− 0.202	0.043	0.203	0.773	0
Community, school	13	0.057	− 0.012	0.125	0.105	< 0.001	76.5
School–home	–	–			–	–	–
Community, home	3	− 0.127	− 0.263	0.01	0.068	0.511	0
Community	8	− 0.052	− 0.14	0.036	0.245	< 0.001	100
Target group							
Children	34	− 0.028	− 0.074	0.018	0.23	< 0.001	99.8
Parent	1	− 0.18	− 0.345	− 0.015	*0.032*		
Children and parents	6	0.034	− 0.023	0.091	0.242	0.891	0
Children and parents and teachers	4	0.03	− 0.135	0.196	0.719	0.058	59.9
Continent							
USA	17	− 0.011	− 0.047	0.025	0.539	0.449	0.4
Europe	6	0.034	− 0.056	0.123	0.46	0.102	45.6
Oceania	21	− 0.032	− 0.09	0.025	0.27	< 0.001	99.9
Asia	1	− 0.1	− 0.155	− 0.045	< *0.001*		
Intervention content^a^							
Education only	4	0.053	− 0.149	0.254	0.609	0.007	75.4
Education as curricula	3	0.026	− 0.138	0.19	0.753	0.015	76.4
Education + change in school environment (such as increased PA or changes in school diet)	15	− 0.071	− 0.128	− 0.014	*0.015*	< 0.001	67
Other community sections	23	− 0.006	− 0.062	0.051	0.841	< 0.001	99.9
Study focus							
Diet + PA	44	− 0.017	− 0.059	0.024	0.415	< 0.001	99.8
PA	1	− 0.1	− 0.155	− 0.045	< *0.001*		
Age-category							
< 5 years old	3	0.072	− 0.063	0.208	0.295	< 0.001	100
5–10 years old	24	− 0.069	− 0.137	− 0.001	*0.046*	< 0.001	78.8
≥ 10 years old	18	0.018	− 0.032	0.067	0.483	< 0.001	67.2
By sample size							
≤ 1000	22	0.015	− 0.03	0.06	0.096	< 0.001	76.8
1000–2000	12	0.015	− 0.03	0.06	0.506	0.142	31.1
≥ 2000	11	0.028	− 0.047	0.103	0.46	< 0.001	99.9
Frequency of intervention							
Daily	11	− 0.029	− 0.105	0.046	0.444	0	99.9
Weekly	18	− 0.013	− 0.068	0.042	0.643	0.002	55.7
Monthly	2	0.067	− 0.075	0.209	0.357	0.687	0
NR^b^	14	− 0.035	− 0.12	0.049	0.412	0	82.7
Duration of intervention (years)							
≤ 1	24	− 0.046	− 0.105	0.012	0.119	< 0.001	68.7
1–2	10	− 0.037	− 0.147	0.072	0.506	< 0.001	86.3
> 2	11	0.033	− 0.042	0.109	0.386	< 0.001	99.9
> 2	1	− 0.14	− 0.277	− 0.003	0.045		
Follow up from baseline (years)							
≤ 1	15	− 0.03	− 0.089	0.029	0.313	0.021	47.4
1–2	14	− 0.016	− 0.108	0.076	0.739	< 0.001	81.1
> 2	16	− 0.02	− 0.083	0.043	0.539	< 0.001	99.9
Sex							
Boys and girls	32	− 0.034	− 0.079	0.012	0.147	< 0.001	99.8
Girls	7	0.011	− 0.084	0.105	0.826	0.272	20.7
Boys	6	0.079	− 0.098	0.257	0.382	0.031	59.4
Quality rating							
Strong	12	0.032	− 0.046	0.109	0.104	< 0.001	75.6
Moderate	16	− 0.054	− 0.118	0.011	0.423	< 0.001	73.9
Weak	17	− 0.024	− 0.092	0.044	0.482	< 0.001	99.9

The nine studies was included as two individual studies [2–4, 14, 21, 23, 33–35], three studies as three individual studies [20, 28, 31], and one study as four individual studies [30]; the significant P-values are presented as italic numbers

*WMD* weighted mean difference, *PA* physical activity

^a^Education, is various training that can be different based on policies, but education as curricula is a unit of instruction in schools that is done as course regularly during the school year

^b^Not reported

**Table 6:** Meta regression analysis for in obesity prevention policies on BMI and BMI-Z_score_

Body mass index (BMI)	Tau^2^	P	95% CI
Estimate of between-study variance	0.020		
By setting/community versus others	0.1461	0.186	− 0.0929, 0.4653
By target group/children versus others	0.1594	0.528	− 0.3282, 0.6312
By country/USA versus others	0.1604	0.728	− 0.3080, 0.4377
By intervention content/education versus others	0.1614	0.923	− 0.4419, 0.4867
By study focus/Diet + PA^a^ versus PA only	0.1402	0.524	− 0.4518, 0.2333
By age/≤ 5 years versus others	0.1586	0.476	− 0.2434, 0.5130
By sample size/≤ 1000 versus others	0.1191	*0.005*	0.0703, 0.3816
By frequency of intervention/ daily versus others	0.16	0.752	− 0.2873, 0.3951
By duration of intervention/≤ 1 year versus others	0.1595	0.322	− 0.1622, 0.4836
By follow-up/≤ 1 year versus others	0.1574	0.285	− 0.1661, 0.5518
By sex/combination of both genders versus others	0.1619	0.589	− 0.4114, 0.2364
By baseline BMI/≤ 18 versus others	0.1604	0.199	− 0.4802, 0.1027
By study quality/strong versus others	0.1572	0.384	− 0.4514, 0.1769
BMI-Z_score_			
Estimate of between-study variance	0.0129		
By setting/community versus others	0.0158	0.173	− 0.0309, 0.1667
By target group/children versus others	0.0168	0.301	− 0.1515, 0.4787
By country/USA versus others	0.0173	0.797	− 0.0854, 0.1106
By intervention content/education versus others	0.0169	0.367	− 0.0951, 0.2525
By study focus/diet + PA versus PA only	0.0173	0.550	− 0.1931, 0.3578
By age/≤ 5 years versus others	0.0164	0.222	− 0.0629, 0.2642
By sample size/≤ 1000 versus others	0.0148	*0.045*	0.0013, 0.1075
By frequency of intervention/daily versus others	0.0174	0.771	− 0.1191, 0.0889
By duration of intervention/≤ 1 year versus others	0.0163	0.253	− 0.1441, 0.0390
By follow-up/≤ 1 year versus others	0.0173	0.906	− 0.1078, 0.0958
By sex/combination of both genders versus others	0.0167	0.210	− 0.1867, 0.0422
By study quality/strong versus others	0.0167	0.268	− 0.1490, 0.0423

The significant P-values are presented as italic numbers

^a^ Physical activity

### Quality assessment of included studies

The Effective Public Health Practice Project Quality Assessment Tool for Quantitative Studies was used for quality assessment of the studies. Study quality [17, 18] was evaluated as “weak” for 15 studies [22, 24, 27–31, 34, 42, 43, 48, 52, 54–56], “moderate” for 10 studies [25, 26, 36, 37, 40, 44, 45, 47, 50, 51], and “strong” for 13 studies [23, 32, 33, 35, 38, 39, 41, 46, 49, 53, 57–59]. Quality assessment results also showed that the average change in BMI or BMI-Z_score_ in the follow-up compared to baseline was 0.5401 and − 0.0054 in the intervention groups and 0.7291 and 0.5401 in the control groups (Additional file 1: Table S3).

### Publication bias

Publication bias was determined using the funnel plot of BMI and BMI-Z_score_ (Additional file 1: Figure S1). Begg's and Egger's regression tests were used to further illustrate publication bias (Additional file 1: Table S7). No evidence of publication bias was seen for BMI in Begg's (*P* = 0.08) or Egger's regression tests (*P* = 0.54) or for BMI-Z_score_ in Begg's (*P* = 0.89) or Egger's regression test (*P* = 0.65).

### Sensitivity analysis

A sensitivity analysis was performed to obtain the effects of individual studies on the BMI-Z_score_ results and the results of the sensitivity analysis is presented as a plot in Additional file 1: Figure S2. By removing the studies of Kremer et al. [45] and de Silva-Sanigorsk et al. [54] a significant change in the results occurred (WMD: − 0.036; CI − 0.068, − 0.005; *P* = 0.025; I^2^ = 72.4; *P* < 0.005). When Sadeghi et al. [42] among boys was also removed, the changes were even more pronounced (WMD: − 0.042; CI − 0.073, − 0.010; *P* = 0.009; I^2^ = 71.5; *P* < 0.001).

## Discussion

This systematic review and meta-analysis is the first, to our knowledge, to evaluate the quantitative effects of various childhood obesity prevention policies on children's BMI and BMI-Z_score_ in an interventional design. There are many systematic reviews or meta-analysis studies that have been performed in specific settings such as schools only [12, 13, 60] or were performed for single-axis interventions such as physical activity only [10, 61], diet only [13] or with limited duration of intervention [62] or follow-up [63, 64] and different age ranges [9, 10, 60, 64]. The current comprehensive meta-analysis evaluated the isolated effects of settings, intervention materials, duration and length of follow up, with a focus on the adiposity-related outcome of BMI or BMI-Z_score_. The key findings of the current study were as follows. First, obesity prevention policies were associated with 0.127 kg/m^2^ reduction in BMI but with no significant change in BMI-Z_score_. Second, there was a nonlinear dose–response association between duration of intervention and reduction in BMI and BMI-Z_score_ in studies with duration of intervention of ≤ 2 years.

In a meta-analysis by Stice et al. [65], no statistically significant effects on prevention or treatment of obesity were reported in a large percentage of studies (79%). In the current meta-analysis childhood obesity prevention policies were associated with 0.127 kg/m^2^ decrease in BMI. This BMI reduction due to weight control programs in the present study was similar to Peirson et al. [63], who assessed 76 studies for normal, overweight and obese children. In contrast in a study by Harris et al., in a systematic review of 18 interventions studies, no significant effects on BMI were found [61]. Another finding in the current study was a small but non-significant change in BMI-Z_score_ in intervention groups (e.g., 0.0054 units’ reduction of BMI-Z_score_ in the intervention vs 0.5401 units’ increase in the control). On the other hand, Peirson et al. [63] found a significant reduction in BMI-Z_score_ in their study. These inconsistencies might be due to differences in inclusion criteria. A nonlinear dose–response association between the duration of intervention (less than 2 years) and decrease in BMI and BMI-Z_score_ indicated long-term duration of intervention reduces the efficacy of weight management policies. As shown in Fig. 2c, for interventions longer than 2 years, the increase in intervention time reduced the mean change in BMI between the intervention and control groups. Consistent with our findings, Stice et al. also found that the weight reducing effects of weight management programs disappeared after a 3-year follow-up, suggesting that short-term obesity prevention programs are more effective than long-term ones in obesity management [65]. These findings were not similar for adults; for example, in a study of adults with an intervention duration that ranged from 6 weeks to 2 years, it was reported that obesity prevention programs could be effective for more than 4 months [66]. Some studies have found no association between the duration of the intervention and weight change [63]. These differences could be due to different populations, age groups, or settings. Stone et al. in a study conducted in Italy to evaluate the effectiveness of the recommended activities in schools, with at least 20 min’ physical activity in a day, reported that less than half of children (49%) took part in the physical activity, while after 7 years follow-up none of the children were engaged in physical activity schedules of more than 20 min [67]. Although we did not show the minimum possible time for the interventions to be effective in this study, the theory of Prochaska and DiClemente [68], recommended that 6 months is the minimum time for stabilizing behavior change involving PA practice. We were not able to assess the long-term sustainability of obesity prevention policies, because there was a limited number of studies that included long-term follow-up after the end of the intervention [54, 69, 70]. From the perspective of the frequency of intervention, optimal frequencies seemed to be daily or weekly schedules, with little effectiveness seen at monthly intervals. It has been established that integration of obesity prevention interventions in the classroom is difficult to achieve [65] and their long-term effectiveness is negligible [67]. Another finding of this study was that school-based programs had the most favorable results in prevention of obesity, which was consistent with the results of some previous studies [64] supporting Centers for Disease Control and Prevention (CDC) [71] and World Health Organization (WHO) [72] recommendations that schools are the best place for obesity prevention in children and adolescents. Wang et al. found that multi-setting trials had beneficial and significant effects compared to single-setting interventions against pediatric obesity [9]. Since most studies of the studies in pediatrics are conducted in schools, further investigations in other settings are indicated to elucidate their effectiveness in pediatric obesity prevention. In our finding, the integration of education alongside changes in the school environment had more favorable results compared with education only. Similarly, Sbruzzi et al. [73] reported that education-only interventions are effective the obesity treatment but not prevention. The heterogeneity of educational materials that are provided in different studies make it difficult to achieve a unique finding about their effectiveness [74]. Most studies (65%) were carried out in either Australia or the United States. Wang et al., in a meta-analysis across high-income countries, found similar results [9]. In subgrouping according to age, reductions in BMI and BMI-Z_score_ were observed in children aged 5–10 years old; similarly, in one study conducted by Peirson et al. in 2013 [63] among 0–18 years old children, beneficial results were observed in the same age range. Richards et al. showed that the strongest effect of PA intervention was found in the youngest children (grade 3 learners compared to the grade 4–6 learners). This was interpreted to be because the intervention promoted PA in the form of playing may have been more attractive and suitable for the younger children [75], or maybe it is because of the ease of interventions in this age groups [76]. On the other hand, high schools and middle schools were more likely to sell competitive foods than were elementary schools [77], which can have a negative impact on the implementation of obesity prevention policies. Finkelstein et al. in their study demonstrated that the consumption of unhealthy foods were high in the high schools children than in elementary school children [78], which is probably due to the fact that the behavior of buying fast food and soft drinks is not fully formed at this age group of children. Finally*,* most of the childhood obesity prevention studies used diet and physical activity combined as an intervention strategy. The result of the current study showed that diet and physical activity-based policies were more effective regarding BMI and BMI-Z_score_ reduction while studies with physical activity-only interventions were not effective. The results of studies by Katz et al. [79], Peirson et al. [63] and Wang et al. [9] found that a combination of diet and physical activity compared to diet-only or physical activity-only interventions had the most favorable results in pediatric obesity prevention. Our sensitivity analysis showed that by removing the studies of Kremer et al. [45], de Silva-Sanigorsk et al. [54] and Sadeghi et al. [42], a significant reduction in BMI-Z_score_ was observed. One of the most important features that these three studies had in common was poor management of selection bias in the quality assessment. As shown by Munafò et al., selection bias can considerably influence observed associations in large-scale cross-sectional studies [80].

## Strengths and limitations

The principal strength of the current study is a comprehensive assessment of obesity prevention policies with an emphasis on different settings, age ranges, and interventional materials and content with BMI and BMI-Z_score_ as target outcomes. We also considered the possible role of the intervention duration, follow-up time and the amount of physical activity by including both randomized and non-randomized controlled clinical trials. Some of the limitations of the current meta-analysis should also be noted; for example, we were not able to obtain the education time and physical activity from all included articles because some of the articles did not specify these. Physical activity and nutrition education interventions are complex and, in each study, different approaches and theories may be used, which in all studies didn’t mention the approach and method of them, therefore, different approaches in educational methods and physical activities made it difficult to classify.

## Conclusion

In conclusion, childhood obesity prevention (a) in school-based policies (b) between the ages of 5–10 years old children, (c) in short-term periods (less than 2 years) at more frequent intervals, (d) with a dual approach of diet and physical activity, can be effective in preventing childhood obesity. These findings can be used by health policymakers and policy providers to apply more effective strategies for obesity prevention in this age group.

## Supplementary information

**Additional file 1: Table S1. **PRISMA checklist. **Table S2**. Search strategies and the number of records according to different electronic database. **Table S3**. Study quality of final studies, assessed by Effective Public Health Practice Project Quality Assessment Tool for quantitative studies. **Table S4.** Full name of studies. **Table S5. **Summary of study findings and additional information of some studies. **Table S6. **The general characteristics of the studies that not include in the meta-analysis. **Table S7.** Publication bias checked by the Begg’s and Egger test in the BMI^a^ and BMI-Z_score_. **Figure S1.** Begg's funnel plot (with pseudo 95% CIs) of the WMD versus the se (WMD) for studies evaluating the effects of obesity preventive policies in children and adolescents and (A) body mass index (BMI) (B) BMI-Z_score_. **Figure S2.** Sensitivity analysis for the effects of childhood obesity prevention policies on BMI-Z_score_.

## Data Availability

The data are available with reasonable request from corresponding authors.

## Abbreviations

BMI: Body mass index
PA: Physical activity
BMI-Z: BMI-Z_score_
PRISMA: Preferred Reporting Items for Systematic Reviews and Meta-Analyses
WMD: Weighted mean difference
CI: Confidence interval
FP: Fractional polynomial

## References

[1] Lindberg L, Danielsson P, Persson M, Marcus C, Hagman E (2020). Association of childhood obesity with risk of early all-cause and cause-specific mortality: a Swedish prospective cohort study. PLoS Med.

[2] World Health Organization, Commission on Ending Childhood Obesity. 2019. https://www.who.int/end-childhood-obesity/en/. Accessed 23 Apr 2020.

[3] Centers for Disease Control, Childhood Obesity Facts. 2020. https://www.cdc.gov/obesity/data/childhood.html. Accessed 26 June 2020.

[4] Simmonds M, Llewellyn A, Owen C, Woolacott N (2016). Predicting adult obesity from childhood obesity: a systematic review and meta-analysis. Obes Rev.

[5] Cawley J (2010). The economics of childhood obesity. Health Aff.

[6] Centers for Disease Control and Prevention, Childhood Obesity Causes &Consequences. 2020. https://www.cdc.gov/obesity/childhood/causes.html. Accessed 1 Nov 2020.

[7] McGuire S, Institute of Medicine (2012). Accelerating progress in obesity prevention: solving the weight of the nation.

[8] Waters E, de Silva‐Sanigorski A, Burford BJ, Brown T, Campbell KJ, Gao Y, Armstrong R, Prosser L, Summerbell CD. Interventions for preventing obesity in children. Cochrane Database Syst Rev. 2011;(12). 10.1002/14651858.CD001871.pub3 22161367

[9] Wang Y, Cai L, Wu Y, Wilson R, Weston C, Fawole O, Bleich SN, Cheskin LJ, Showell NN, Lau B (2015). What childhood obesity prevention programmes work? A systematic review and meta-analysis. Obes Rev.

[10] Finch M, Jones J, Yoong S, Wiggers J, Wolfenden L (2016). Effectiveness of centre-based childcare interventions in increasing child physical activity: a systematic review and meta-analysis for policymakers and practitioners. Obes Rev.

[11] Moores C, Bell L, Miller J, Damarell R, Matwiejczyk L, Miller M (2018). A systematic review of community-based interventions for the treatment of adolescents with overweight and obesity. Obes Rev.

[12] Levinson J, Kohl K, Baltag V, Ross DA (2019). Investigating the effectiveness of school health services delivered by a health provider: a systematic review of systematic reviews. PLoS ONE.

[13] Micha R, Karageorgou D, Bakogianni I, Trichia E, Whitsel LP, Story M, Penalvo JL, Mozaffarian D (2018). Effectiveness of school food environment policies on children’s dietary behaviors: a systematic review and meta-analysis. PLoS ONE.

[14] Hale I. Obesity prevention: are we missing the (conception to infancy) window?, Royal College of General Practitioners. 2018. pp. 262–3. 10.3399/bjgp18X696269 PMC6002001 29853573

[15] Aziz Z, Absetz P, Oldroyd J, Pronk NP, Oldenburg B (2015). A systematic review of real-world diabetes prevention programs: learnings from the last 15 years. Implement Sci.

[16] Moher D, Liberati A, Tetzlaff J, Altman DG, Group, P (2009). Preferred reporting items for systematic reviews and meta-analyses: the PRISMA statement. PLoS Med.

[17] Deeks JJ, Dinnes J, D'Amico R, Sowden AJ, Sakarovitch C, Song F, Petticrew M, Altman D (2003). Evaluating non-randomised intervention studies. Health Technol Assess.

[18] Thomas B, Ciliska D, Dobbins M, Micucci S (2004). A process for systematically reviewing the literature: providing the research evidence for public health nursing interventions. Worldviews Evid Based Nurs.

[19] Higgins J, Thompson S (2002). Quantifying heterogeneity in a meta-analysis. Stat Med.

[20] Walter S, Yao X (2007). Effect sizes can be calculated for studies reporting ranges for outcome variables in systematic reviews. J Clin Epidemiol.

[21] Fan J, Gijbels I (1996). Local polynomial modelling and its applications: monographs on statistics and applied probability 66.

[22] Wang Z, Xu F, Ye Q, Tse LA, Xue H, Tan Z, Leslie E, Owen N, Wang Y (2018). Childhood obesity prevention through a community-based cluster randomized controlled physical activity intervention among schools in china: the health legacy project of the 2nd world summer youth olympic Games (YOG-Obesity study). Int J Obes.

[23] Leme ACB, Baranowski T, Thompson D, Nicklas T, Philippi ST (2018). Sustained impact of the “Healthy Habits, Healthy Girls—Brazil” school-based randomized controlled trial for adolescents living in low-income communities. Prev Med Rep.

[24] Lubans DR, Smith JJ, Plotnikoff RC, Dally KA, Okely AD, Salmon J, Morgan PJ (2016). Assessing the sustained impact of a school-based obesity prevention program for adolescent boys: the ATLAS cluster randomized controlled trial. Int J Behav Nutr Phys Act.

[25] Hollis JL, Sutherland R, Campbell L, Morgan PJ, Lubans DR, Nathan N, Wolfenden L, Okely AD, Davies L, Williams A, Cohen KE, Oldmeadow C, Gillham K, Wiggers J (2016). Effects of a 'school-based' physical activity intervention on adiposity in adolescents from economically disadvantaged communities: secondary outcomes of the 'Physical Activity 4 Everyone' RCT. Int J Obes.

[26] Smith JJ, Morgan PJ, Plotnikoff RC, Dally KA, Salmon J, Okely AD, Finn TL, Lubans DR (2014). Smart-phone obesity prevention trial for adolescent boys in low-income communities: The ATLAS RCT. Pediatrics.

[27] Lubans DR, Morgan PJ, Okely AD, Dewar D, Collins CE, Batterham M, Callister R, Plotnikoff RC (2012). Preventing obesity among adolescent girls: one-year outcomes of the nutrition and enjoyable activity for teen girls (NEAT Girls) cluster randomized controlled trial. Arch Pediatr Adolesc Med.

[28] Millar L, Kremer P, de Silva-Sanigorski A, McCabe MP, Mavoa H, Moodie M, Utter J, Bell C, Malakellis M, Mathews L, Roberts G, Robertson N, Swinburn BA (2011). Reduction in overweight and obesity from a 3-year community-based intervention in Australia: the 'It's Your Move!' project. Obes Rev.

[29] Llargues E, Franco R, Recasens A, Nadal A, Vila M, Pérez MJ, Manresa JM, Recasens I, Salvador G, Serra J, Roure E, Castells C (2011). Assessment of a school-based intervention in eating habits and physical activity in school children: The AVall study. J Epidemiol Community Health.

[30] Salcedo Aguilar F, Martinez-Vizcaino V, Sanchez Lopez M, Solera Martinez M, Franquelo Gutierrez R, Serrano Martinez S, Lopez-Garcia E, Rodriguez-Artalejo F (2010). Impact of an after-school physical activity program on obesity in children. J Pediatr.

[31] Neumark-Sztainer DR, Friend SE, Flattum CF, Hannan PJ, Story MT, Bauer KW, Feldman SB, Petrich CA (2010). New moves-preventing weight-related problems in adolescent girls: a group-randomized study. Am J Prev Med.

[32] Group, H.S (2010). A school-based intervention for diabetes risk reduction. N Engl J Med.

[33] Dzewaltowski DA, Rosenkranz RR, Geller KS, Coleman KJ, Welk GJ, Hastmann TJ, Milliken GA (2010). HOP'N after-school project: an obesity prevention randomized controlled trial. Int J Behav Nutr Phys Act.

[34] Donnelly JE, Greene JL, Gibson CA, Smith BK, Washburn RA, Sullivan DK, DuBose K, Mayo MS, Schmelzle KH, Ryan JJ (2009). Physical Activity Across the Curriculum (PAAC): a randomized controlled trial to promote physical activity and diminish overweight and obesity in elementary school children. Prev Med Int J Devoted Pract Theory.

[35] Taylor R, McAuley K, Barbezat W, Farmer V, Williams S, Mann J (2008). Two-year follow-up of an obesity prevention initiative in children: the APPLE project 1-3. Am J Clin Nutr.

[36] Martínez Vizcaíno V, Salcedo Aguilar F, Franquelo Gutiérrez R, Solera Martínez M, Sánchez López M, Serrano Martínez S, López García E, Rodríguez Artalejo F (2008). Assessment of an after-school physical activity program to prevent obesity among 9- to 10-year-old children: A cluster randomized trial. Int J Obes.

[37] Foster GD, Sherman S, Borradaile KE, Grundy KM, Vander Veur SS, Nachmani J, Karpyn A, Kumanyika S, Shults J (2008). A policy-based school intervention to prevent overweight and obesity. Pediatrics.

[38] Bell L, Ullah S, Leslie E, Magarey A, Olds T, Ratcliffe J, Chen G, Miller M, Jones M, Cobiac L (2019). Changes in weight status, quality of life and behaviours of South Australian primary school children: results from the Obesity Prevention and Lifestyle (OPAL) community intervention program. BMC Public Health.

[39] Santiago Felipe G, Rafael Casas E, Subirana I, Serra-Majem L, Torrent MF, Homs C, Rowaedh Ahmed B, Estrada L, Fíto M, Schröder H (2018). Effect of a community-based childhood obesity intervention program on changes in anthropometric variables, incidence of obesity, and lifestyle choices in Spanish children aged 8 to 10 years. Eur J Pediatr.

[40] Novotny R, Davis J, Butel J, Boushey CJ, Fialkowski MK, Nigg CR, Braun KL, Leon Guerrero RT, Coleman P, Bersamin A, Areta AAR, Barber LR, Belyeu-Camacho T, Greenberg J, Fleming T, Dela Cruz-Talbert E, Yamanaka A, Wilkens LR (2018). Effect of the children's healthy living program on young child overweight, obesity, and acanthosis nigricans in the US-affiliated pacific region: a randomized clinical trial. JAMA Netw Open.

[41] Adab P, Pallan MJ, Lancashire ER, Hemming K, Frew E, Barrett T, Bhopal R, Cade JE, Canaway A, Clarke JL, Daley A, Deeks JJ, Duda JL, Ekelund U, Gill P, Griffin T, McGee E, Hurley K, Martin J, Parry J, Passmore S, Cheng KK (2018). Effectiveness of a childhood obesity prevention programme delivered through schools, targeting 6 and 7 year olds: cluster randomised controlled trial (WAVES study). BMJ (Online).

[42] Sadeghi B, Kaiser L, Schaefer S, Tseregounis I, Martinez L, Gomez-Camacho R, de la Torre A (2017). Multifaceted community-based intervention reduces rate of BMI growth in obese Mexican-origin boys. Pediatr Obes.

[43] Swinburn B, Malakellis M, Moodie M, Waters E, Gibbs L, Millar L, Herbert J, Virgo-Milton M, Mavoa H, Kremer P, De Silva-Sanigorski A (2014). Large reductions in child overweight and obesity in intervention and comparison communities 3 years after a community project. Pediatr Obes.

[44] Pettman T, Magarey A, Mastersson N, Wilson A, Dollman J (2014). Improving weight status in childhood: results from the eat well be active community programs. Int J Public Health.

[45] Kremer P, Waqa G, Vanualailai N, Schultz JT, Roberts G, Moodie M, Mavoa H, Malakellis M, McCabe MP, Swinburn BA (2011). Reducing unhealthy weight gain in Fijian adolescents: results of the Healthy Youth Healthy Communities study. Obes Rev.

[46] Fotu K, Millar L, Mavoa H, Kremer P, Moodie M, Snowdon W, Utter J, Vivili P, Schultz J, Malakellis M (2011). Outcome results for the Ma'alahi Youth Project, a Tongan community-based obesity prevention programme for adolescents. Obes Rev.

[47] Sanigorski AM, Bell AC, Kremer PJ, Cuttler R, Swinburn BA (2008). Reducing unhealthy weight gain in children through community capacity-building: results of a quasi-experimental intervention program, Be Active Eat Well. Int J Obes.

[48] Romon M, Lommez A, Tafflet M, Basdevant A, Oppert JM, Bresson JL, Ducimetiére P, Charles MA, Borys JM (2009). Downward trends in the prevalence of childhood overweight in the setting of 12-year school- and community-based programmes. Public Health Nutr.

[49] Crespo NC, Elder JP, Ayala GX, Slymen DJ, Campbell NR, Sallis JF, McKenzie TL, Baquero B, Arredondo EM (2012). Results of a multi-level intervention to prevent and control childhood obesity among Latino children: the Aventuras Para Niños Study. Ann Behav Med.

[50] Gentile DA, Welk G, Eisenmann JC, Reimer RA, Walsh DA, Russell DW, Callahan R, Walsh M, Strickland S, Fritz K (2009). Evaluation of a multiple ecological level child obesity prevention program: switch what you Do, View, and Chew. BMC Med.

[51] Economos CD, Hyatt RR, Goldberg JP, Must A, Naumova EN, Collins JJ, Nelson ME (2007). A community intervention reduces BMI z-score in children: Shape up somerville first year results. Obesity.

[52] Wong WW, Ortiz CL, Stuff JE, Mikhail C, Lathan D, Moore LA, Alejandro ME, Butte NF, Smith EO (2016). A community-based healthy living promotion program improved self-esteem among minority children. J Pediatr Gastroenterol Nutr.

[53] Johnson BA, Kremer PJ, Swinburn BA, De Silva-Sanigorski AM (2012). Multilevel analysis of the Be Active Eat Well intervention: environmental and behavioural influences on reductions in child obesity risk. Int J Obes.

[54] de Silva-Sanigorski AM, Bell AC, Kremer P, Nichols M, Crellin M, Smith M, Sharp S, de Groot F, Carpenter L, Boak R (2010). Reducing obesity in early childhood: results from Romp & Chomp, an Australian community-wide intervention program. Am J Clin Nutr.

[55] Taylor RW, McAuley KA, Barbezat W, Strong A, Williams SM, Mann JI (2007). APPLE Project: 2-y findings of a community-based obesity prevention program in primary school-age children. Am J Clin Nutr.

[56] de Henauw S, Huybrechts I, de Bourdeaudhuij I, Bammann K, Barba G, Lissner L, Mårild S, Molnár D, Moreno LA, Pigeot I, Tornaritis M, Veidebaum T, Verbestel V, Ahrens W (2015). Effects of a community-oriented obesity prevention programme on indicators of body fatness in preschool and primary school children. Main results from the IDEFICS study. Obes Rev.

[57] Elder JP, Crespo NC, Corder K, Ayala GX, Slymen DJ, Lopez NV, Moody JS, McKenzie TL (2014). Childhood obesity prevention and control in city recreation centres and family homes: the MOVE/me Muevo Project. Pediatr Obes.

[58] Eno Persson J, Bohman B, Tynelius P, Rasmussen F, Ghaderi A (2018). Prevention of childhood obesity in child health services: follow-up of the PRIMROSE trial. Child Obes.

[59] Hammersley ML, Okely AD, Batterham MJ, Jones RA (2019). An internet-based childhood obesity prevention program (TIMe2bhealthy) for parents of preschool-aged children: randomized controlled trial. J Med Internet Res.

[60] Verjans-Janssen SRB, van de Kolk I, Van Kann DHH, Kremers SPJ, Gerards SMPL (2018). Effectiveness of school-based physical activity and nutrition interventions with direct parental involvement on children's BMI and energy balance-related behaviors—a systematic review. PLoS ONE.

[61] Harris KC, Kuramoto LK, Schulzer M, Retallack JE (2009). Effect of school-based physical activity interventions on body mass index in children: a meta-analysis. CMAJ.

[62] Lim S, Hill B, Teede HJ, Moran LJ, O’Reilly S (2020). An evaluation of the impact of lifestyle interventions on body weight in postpartum women: a systematic review and meta-analysis. Obes Rev.

[63] Peirson L, Fitzpatrick-Lewis D, Morrison K, Ciliska D, Kenny M, Usman Ali M, Raina P (2015). Prevention of overweight and obesity in children and youth: a systematic review and meta-analysis. CMAJ Open.

[64] Bleich SN, Segal J, Wu Y, Wilson R, Wang Y (2013). Systematic review of community-based childhood obesity prevention studies. Pediatrics.

[65] Stice E, Shaw H, Marti CN (2006). A Meta-Analytic Review of Obesity Prevention Programs for Children and Adolescents: The Skinny on Interventions That Work. Psychol Bull.

[66] Hebden L, Chey T, Allman-Farinelli M (2012). Lifestyle intervention for preventing weight gain in young adults: a systematic review and meta-analysis of RCTs. Obes Rev.

[67] Stone MR, Faulkner GE, Zeglen-Hunt L, Bonne JC (2012). The Daily Physical Activity (DPA) policy in Ontario: is it working? An examination using accelerometry-measured physical activity data. Can J Public Health.

[68] Prochaska JO, DiClemente CC (1992). Stages of change in the modification of problem behaviors. Prog Behav Modif.

[69] Lazorick S, Fang X, Crawford Y (2016). The MATCH program: long-term obesity prevention through a middle school based intervention. Child Obes.

[70] Rush E, McLennan S, Obolonkin V, Vandal AC, Hamlin M, Simmons D, Graham D (2014). Project Energize: whole-region primary school nutrition and physical activity programme; evaluation of body size and fitness 5 years after the randomised controlled trial. Br J Nutr.

[71] CDC, CDC Healthy Schools. 2019. https://www.cdc.gov/healthyschools/health_and_academics/index.htm. Accessed 26 June 2020.

[72] World Health Organization, Global Strategy on Diet, Physical Activity and Health. 2020. https://www.who.int/dietphysicalactivity/childhood_schools/en/. Accessed 26 June 2020.

[73] Sbruzzi G, Eibel B, Barbiero SM, Petkowicz RO, Ribeiro RA, Cesa CC, Martins CC, Marobin R, Schaan CW, Souza WB (2013). Educational interventions in childhood obesity: a systematic review with meta-analysis of randomized clinical trials. Prev Med.

[74] Shrewsbury VA, Nguyen B, O'Connor J, Steinbeck KS, Lee A, Hill AJ, Shah S, Kohn MR, Torvaldsen S, Baur LA (2011). Short-term outcomes of community-based adolescent weight management: the Loozit® study. BMC Pediatr.

[75] Richards J, Foster C (2013). Sport-for-development interventions: whom do they reach and what is their potential for impact on physical and mental health in low-income countries?. J Phys Activity Health.

[76] Stock S, Miranda C, Evans S, Plessis S, Ridley J, Yeh S, Chanoine J-P (2007). Healthy Buddies: a novel, peer-led health promotion program for the prevention of obesity and eating disorders in children in elementary school. Pediatrics.

[77] Story M, Nanney MS, Schwartz MB (2009). Schools and obesity prevention: creating school environments and policies to promote healthy eating and physical activity. Milbank Q.

[78] Finkelstein DM, Hill EL, Whitaker RC (2008). School food environments and policies in US public schools. Pediatrics.

[79] Katz DL, O'Connell M, Njike VY, Yeh M-C, Nawaz H (2008). Strategies for the prevention and control of obesity in the school setting: systematic review and meta-analysis. Int J Obes.

[80] Munafò MR, Tilling K, Taylor AE, Evans DM, Davey Smith G (2018). Collider scope: when selection bias can substantially influence observed associations. Int J Epidemiol.

