# Supplementary material for: The effectiveness of pediatric obesity prevention policies: a comprehensive systematic review and dose–response meta-analysis of controlled clinical trials
Source: J Transl Med. 2020 Dec 14;18:480. doi: 10.1186/s12967-020-02640-1 (PMC7734784; doi:10.1186/s12967-020-02640-1)
Supplement: Supplementary file 1 — Additional file 1: Table S1. PRISMA checklist. Table S2. Search strategies and the number of records according to different electronic database. Table S3. Study quality of final studies, assessed by Effective Public Health Practice Project Quality Assessment Tool for quantitative studies. Table S4. Full name of studies. Table S5. Summary of study findings and additional information of some studies. Table S6. The general characteristics of the studies that not include in the meta-analysis. Table S7. Publication bias checked by the Begg’s and Egger test in the BMIa and BMI-Zscore. Figure S1. Begg's funnel plot (with pseudo 95% CIs) of the WMD versus the se (WMD) for studies evaluating the effects of obesity preventive policies in children and adolescents and (A) body mass index (BMI) (B) BMI-Zscore. Figure S2. Sensitivity analysis for the effects of childhood obesity prevention policies on BMI-Zscore. [file 12967_2020_2640_MOESM1_ESM.docx]

**Title: The effectiveness of pediatric obesity prevention policies: A comprehensive systematic review and dose-response meta-analysis of controlled clinical trials**

Authors: Shahnaz Taghizadeh ^1^, Mahdieh Abbasalizad Farhangi ^2*^

^1^ Molecular Medicine Research Center, Tabriz University of Medical sciences, Tabriz, Iran.

^2^ Drug Applied Research Center, Tabriz University of Medical Sciences, Tabriz-Iran.

* **Correspondence**: Attar Nishabouri St., Tabriz, I. R. Iran; Postal code: 5166614711, POBOX: 14711; Tel: +98-41-33362117, Fax: +98-41-33340634; email: [abbasalizad_m@yahoo.com](mailto:abbasalizad_m@yahoo.com)

**Additional file**

| **Table S1.** PRISMA Checklist | | | | | |
| --- | --- | --- | --- | --- | --- |
| ^{Moher, 1996 #218}^**Section/topic** | **#** | | | **Checklist item** | **Reported on page #** |
| **TITLE** | | | | |  |
| Title | 1 | | | Identify the report as a systematic review, meta-analysis, or both. | 1 |
| **ABSTRACT** | | | | |  |
| Structured summary | 2 | | | Provide a structured summary including, as applicable: background; objectives; data sources; study eligibility criteria, participants, and interventions; study appraisal and synthesis methods; results; limitations; conclusions and implications of key findings; systematic review registration number. | 2 |
| **INTRODUCTION** | | | | |  |
| Rationale | 3 | | | Describe the rationale for the review in the context of what is already known. | 3 |
| Objectives | 4 | | | Provide an explicit statement of questions being addressed with reference to participants, interventions, comparisons, outcomes, and study design (PICOS). | 3 |
| **METHODS** | | | | |  |
| Protocol and registration | 5 | Indicate if a review protocol exists, if and where it can be accessed (e.g., Web address), and, if available, provide registration information including registration number. | | | 4 |
| Eligibility criteria | 6 | Specify study characteristics (e.g., PICOS, length of follow-up) and report characteristics (e.g., years considered, language, publication status) used as criteria for eligibility, giving rationale. | | | 5 |
| Information sources | 7 | Describe all information sources (e.g., databases with dates of coverage, contact with study authors to identify additional studies) in the search and date last searched. | | | 4 |
| Search | 8 | Present full electronic search strategy for at least one database, including any limits used, such that it could be repeated. | | | 4 |
| Study selection | 9 | State the process for selecting studies (i.e., screening, eligibility, included in systematic review, and, if applicable, included in the meta-analysis). | | | 4 |
| Data collection process | 10 | Describe method of data extraction from reports (e.g., piloted forms, independently, in duplicate) and any processes for obtaining and confirming data from investigators. | | | 5 |
| Data items | 11 | List and define all variables for which data were sought (e.g., PICOS, funding sources) and any assumptions and simplifications made. | | | 4-5 |
| Risk of bias in individual studies | 12 | Describe methods used for assessing risk of bias of individual studies (including specification of whether this was done at the study or outcome level), and how this information is to be used in any data synthesis. | | | 5 |
| Summary measures | 13 | State the principal summary measures (e.g., risk ratio, difference in means). | | | 5 |
| Synthesis of results | 14 | Describe the methods of handling data and combining results of studies, if done, including measures of consistency (e.g., I^2^) for each meta-analysis. | | | 5 |
| Risk of bias across studies | 15 | | Specify any assessment of risk of bias that may affect the cumulative evidence (e.g., publication bias, selective reporting within studies). | | 5 |
| Additional analyses | 16 | | Describe methods of additional analyses (e.g., sensitivity or subgroup analyses, meta-regression), if done, indicating which were pre-specified. | | 5 |
| **RESULTS** | | | | |  |
| Study selection | 17 | | Give numbers of studies screened, assessed for eligibility, and included in the review, with reasons for exclusions at each stage, ideally with a flow diagram. | | 6 |
| Study characteristics | 18 | | For each study, present characteristics for which data were extracted (e.g., study size, PICOS, follow-up period) and provide the citations. | | 6,7 |
| Risk of bias within studies | 19 | | Present data on risk of bias of each study and, if available, any outcome level assessment (see item 12). | | 9 |
| Results of individual studies | 20 | | For all outcomes considered (benefits or harms), present, for each study: (a) simple summary data for each intervention group (b) effect estimates and confidence intervals, ideally with a forest plot. | | 8 |
| Synthesis of results | 21 | | Present results of each meta-analysis done, including confidence intervals and measures of consistency. | | 8 |
| Risk of bias across studies | 22 | | Present results of any assessment of risk of bias across studies (see Item 15). | | 8,9 |
| Additional analysis | 23 | | Give results of additional analyses, if done (e.g., sensitivity or subgroup analyses, meta-regression [see Item 16]). | | 8,9 |
| **DISCUSSION** | | | | |  |
| Summary of evidence | 24 | | Summarize the main findings including the strength of evidence for each main outcome; consider their relevance to key groups (e.g., healthcare providers, users, and policy makers). | | 10,12 |
| Limitations | 25 | | Discuss limitations at study and outcome level (e.g., risk of bias), and at review-level (e.g., incomplete retrieval of identified research, reporting bias). | | 12 |
| Conclusions | 26 | | Provide a general interpretation of the results in the context of other evidence, and implications for future research. | | 12 |
| **FUNDING** | | | | |  |
| Funding | 27 | | Describe sources of funding for the systematic review and other support (e.g., supply of data); role of funders for the systematic review. | | 13 |

| **Table S2**. Search strategies and the number of records according to different electronic database | | |
| --- | --- | --- |
| **search strategy** | **Database** | **Num. of records** |
| Search (((((("Policy"[Mesh] OR "Nutrition Policy"[Mesh] OR "Policy Making"[Mesh] OR "Health Policy"[Mesh])) OR Program[Title/Abstract]) OR Strategy[Title/Abstract])) AND ((("Obesity"[Mesh] OR "Pediatric Obesity"[Mesh] OR "Obesity, Abdominal"[Mesh] OR "Obesity Management"[Mesh])) OR "Overweight"[Mesh])) AND ((((((("Child"[Mesh] OR "Child, Preschool"[Mesh])) OR "Pediatrics"[Mesh]) OR ("Adolescent"[Mesh] OR "Adolescent Health"[Mesh])) OR paediatric[Title/Abstract]) OR teenager[Title/Abstract]) OR youth[Title/Abstract]) | SCOPUS | 20708 |
|  | PUBMED | 5301 |
|  | EMBASE | 4675 |

| **Table S3**. Study quality of final studies, assessed by Effective Public Health Practice Project Quality Assessment Tool for quantitative studies | | | | | | | |
| --- | --- | --- | --- | --- | --- | --- | --- |
| **First Author /Year** | **Component Ratings** | | | | | | **Global Rating** |
|  | **Selection Bias** | **Study Design** | **Blinding** | **Confounders** | **Data Collection Method** | **Withdrawals & Dropouts** |  |
| Wang Z / 2018 (1) | strong | strong | strong | weak | strong | weak | weak |
| Leme ACB /2018 (2) | strong | strong | strong | strong | strong | strong | strong |
| Lubans DR /2016 (3) | strong | strong | moderate | weak | moderate | weak | weak |
| Hollis JL /2016 (4) | moderate | strong | moderate | weak | moderate | strong | moderate |
| Smith JJ /2014 (5) | strong | strong | strong | weak | strong | strong | moderate |
| Lubans DR /2012 (6) | strong | strong | weak | weak | strong | strong | weak |
| Millar L /2011 (7) | moderate | strong | weak | weak | strong | weak | weak |
| Llargues E /2011 (8) | strong | strong | weak | strong | strong | weak | weak |
| Salcedo Aguilar F /2010 (9) | moderate | strong | weak | N/A | strong | weak | weak |
| Neumark-Sztainer DR /2010 (10) | moderate | strong | weak | moderate | weak | weak | weak |
| Group HS / 2010 (11) | strong | strong | strong | strong | strong | strong | strong |
| Dzewaltowski DA /2010 (12) | strong | strong | strong | moderate | strong | strong | strong |
| Donnelly JE / 2009 (13) | moderate | strong | moderate | weak | strong | weak | weak |
| Taylor R /2008 (14) | moderate | strong | moderate | moderate | strong | strong | strong |
| Martínez Vizcaíno V / 2008 (15) | strong | strong | weak | moderate | strong | moderate | moderate |
| Foster GD / 2008 (16) | moderate | strong | moderate | moderate | strong | weak | moderate |
| Bell L /2019 (17) | moderate | strong | moderate | strong | strong | strong | strong |
| Santiago Felipe G /2018 (18) | strong | strong | moderate | strong | strong | strong | strong |
| Novotny R / 2018 (19) | strong | weak | strong | strong | strong | strong | moderate |
| Adab P /2018 (20) | moderate | strong | moderate | strong | strong | strong | strong |
| Sadeghi B / 2017 (21) | weak | strong | strong | weak | strong | moderate | weak |
| Swinburn B /2014 (22) | moderate | strong | moderate | weak | strong | weak | weak |
| Pettman T / 2014 (23) | weak | strong | moderate | moderate | strong | moderate | moderate |
| Kremer P /2011 (24) | weak | strong | moderate | moderate | strong | strong | moderate |
| Fotu K / 2011 (25) | strong | strong | strong | strong | strong | strong | strong |
| Sanigorski AM /2008 (26) | strong | strong | weak | moderate | strong | moderate | moderate |
| Romon M /2009 (27) | moderate | strong | weak | weak | strong | weak | weak |
| Crespo NC /2012(28) | moderate | strong | strong | strong | strong | strong | strong |
| Gentile DA / 2009 (29) | moderate | strong | weak | moderate | moderate | moderate | moderate |
| Economos CD /2007 (30) | weak | strong | moderate | N/A | strong | strong | moderate |
| Wong WW /2016 (31) | weak | strong | moderate | weak | strong | weak | weak |
| Johnson BA / 2012 (32) | strong | strong | moderate | moderate | strong | strong | strong |
| de Silva-Sanigorski AM /  2010 (33) | weak | weak | moderate | weak | strong | weak | weak |
| Taylor RW /2007 (34) | moderate | strong | weak | moderate | strong | weak | weak |
| de Henauw S /2015 (35) | strong | strong | weak | strong | weak | strong | weak |
| Elder JP /2014 (36) | strong | strong | moderate | strong | strong | moderate | strong |
| Eno Persson J /2018 (37) | strong | strong | moderate | moderate | strong | moderate | strong |
| Hammersley ML /2019 (38) | strong | strong | strong | strong | moderate | moderate | strong |
| **Summary [n (%)]** | | | | | | | |
| Strong | 18 (20.5) | 36 (40.9) | 10 (11.4) | 12 (13.6) | 32 (36.4) | 17 (19.3) | 13 (14.8) |
| Moderate | 14 (15.9) | - | 17 (19.3) | 12 (13.6) | 4 (4.5) | 8 (9.1) | 10 (11.4) |
| Weak | 6 (6.8) | 2 (2.3) | 11 (12.5) | 12 (13.6) | 2 (2.3) | 13 (14.8) | 15 (17.0) |
| N/A | - | - | - | 2 (2.3) | - | - | - |
| N/A, not applicable; strong = no weak ratings; Moderate = 1 weak rating; weak = ≥ 2 weak ratings | | | | | | | |

| **Table S4.** Full name of studies | |
| --- | --- |
| YOG-Obesity study | Youth Olympic Games - Obesity study |
| H3G-Brazil | Healthy Habits, Healthy Girls – Brazil |
| ATLAS | Active Teen Leaders Avoiding Screen-time |
| PA4E1 | Physical Activity 4 Everyone |
| ATLAS | Active Teen Leaders Avoiding Screen-time |
| NEAT Girl | Nutrition and enjoyable activity for teen girls |
| IYM | It’s Your Move! |
| AVall | Assessment of a School-based Intervention in Eating Habits and Physical Activity in Schoolchildren |
| MOVI | After-School Physical Activity Program on Obesity in Children |
| New Moves | Preventing Weight-Related Problems in Adolescent Girls |
| School-based program on risk factors for DM | school-based program on risk factors for DM |
| HOP’N | Healthy Opportunities for Physical Activity and Nutrition |
| PAAC | Physical Activity Across the Curriculum |
| APPLE | A Pilot Programme for Lifestyle and Exercise |
| Movi | non-competitive recreational physical activity program |
| SNPI | School Nutrition Policy Initiative |
| OPAL | Obesity Prevention and Lifestyle |
| TCHP | Thao-Child Health Program |
| Children’s Healthy Living Program | Children’s Healthy Living Program |
| WAVES | West Midlands ActiVe lifestyle and healthy Eating in School children |
| NSFS | Niños Sanos, Familia Sana |
| BAEW | Be Active Eat Well |
| ewba | eat well be active Community Programs |
| HYHC | Healthy Youth Healthy Communities |
| MYP | Ma’alahi Youth Project |
| BAEW | Be Active Eat Well |
| FLVS | Fleurbaix– Laventie Ville Sante |
| APN | Aventuras para Niños |
| Switch& what you Do, View, and Chew | Switch& what you Do, View, and Chew |
| SUS | Shape Up Somerville |
| Healthy Kids Houston | Healthy Kids Houston |
| BAEW | Be Active Eat Well |
| Romp & Chomp | Romp & Chomp |
| APPLE | A Pilot Programme for Lifestyle and Exercise |
| IDEFICS | Identification and prevention of dietary- and lifestyle-induced health effects in children and infants |
| MOVE | Muevo Family Health Program |
| PRIMROSE | Prevention of Childhood Obesity in Child Health Services |
| Time2bHealthy | Time to be Healthy |
| Daily Mile | Run or walk outside for 15 min (~1 mile) |
| Fun ‘n healthy in Moreland | Fun ‘n healthy in Moreland |
| Let's Go! 5-2-1-0 | Let's Go! 5-2-1-0 pediatric obesity intervention program |
| MATCH | Motivating Adolescents with Technology to CHOOSE Health |
| Healthy Buddies | Healthy Buddies |
| Project Energize | whole-region primary school nutrition and physical activity programme |
| Gold Medal Schools Program | Gold Medal Schools Program |
| Mebane on the Move | Mebane on the Move |
| TOTS | Toddler overweight and tooth decay prevention study |
| MA-CORD | Massachusetts Childhood Obesity Research Demonstration |
| CCHP | Child Care Health Program |

| **Table S5.** Summary of study findings and additional information of some studies | | | |
| --- | --- | --- | --- |
| **First Author / Year (reference)** | **Economic status of country** | **Additional information** | **Main findings** |
| Wang Z / 2018 (1) | NR ^b^ | - | ↓ BMI in the IN ^c^ groups compared to the CN^d^ groups (p<0.05)  ↓ BMI-Z in the IN groups compared to the CN groups (p<0.05) |
| Leme ACB /2018 (2) | Low income | The duration of intervention was 6 months and fallowed after 6 months, Immediately After the End of the Intervention (IAEI) and 12 months from baseline. This program was run only on girls | No significant change in BMI and BMI-Z^f^ between the IN groups compared to the CN groups after 6 and 12 months |
| Lubans DR /2016 (3) | Low income | The duration of intervention was 8 months and fallowed after 8(IAEI) and 18 months from baseline. This program was run only on boys | No significant change in BMI and BMI-Z between the IN groups compared to the CN groups after 8 and 18 months from baseline |
| Hollis JL /2016 (4) | Low income | The program lasted 2 years. Fallowed –up once in the middle of the program, one year after the baseline (total increase of PA ^h^ 70 hrs.) and again 2 years after the baseline (total increase of PA 156 hrs.). | ↓ BMI in the IN groups compared to the CN groups (p<0.05)  after 1 and 2 years  ↓ BMI-Z in the IN groups compared to the CN groups (p<0.05) after 1 and 2 years |
| Smith JJ /2014 (5) | Low income | This program was run only on boys | No significant change in BMI between the IN groups compared to the CN groups |
| Lubans DR /2012 (6) | Low income | This program was run only on girls | No significant change in BMI and BMI-Z between the IN groups compared to the CN groups |
| Millar L /2011 (7) | Low income | - | ↓ BMI in the IN groups compared to the CN groups (p<0.05)  ↓ BMI-Z in the IN groups compared to the CN groups (p<0.05) |
| Llargues E /2011 (8) | Low income | - | No significant change in BMI between the IN groups compared to the CN groups |
| Salcedo Aguilar F /2010 (9) | NR | The duration of intervention was 12 months (total increase of PA 234 hrs.), and 20 months (total increase of PA 468 hrs.) and analyzed separately in the girls and the boys | No significant change in BMI between the IN groups compared to the CN groups in the boys and the girls |
| Neumark-Sztainer DR /2010 (10) | Low income | The duration of intervention was 4 months and fallowed after 4(IAEI) and 9 months from baseline. This program was run only on girls | No significant change in BMI between the IN groups compared to the CN groups after 4 and 9 months from baseline |
| Group HS / 2010 (11) | Low income | - | ↓ BMI in the IN groups compared to the CN groups (p<0.05) |
| Dzewaltowski DA /2010 (12) | NR | - | No significant change in BMI and BMI-Z between the IN groups compared to the CN groups |
| Donnelly JE / 2009 (13) | NR | - | No significant change in BMI between the IN groups compared to the CN groups |
| Taylor R /2008 (14) | NR | The duration of intervention was 12 months and fallowed after 12(IAEI) and 30 months from baseline | BMI was one of the outcoms, but its changes were not significantly investigated  ↓ BMI-Z in the IN groups compared to the CN groups (p<0.05) |
| Martínez Vizcaíno V / 2008 (15) | NR | Analyzed separately in the girls and the boys | No significant change in BMI between the IN groups compared to the CN groups |
| Foster GD / 2008 (16) | Low income | - | No significant change in BMI-Z between the IN groups compared to the CN groups |
| Bell L /2019 (17) | Low income | - | No significant change in BMI between the IN groups compared to the CN groups |
| Santiago Felipe G /2018 (18) | NR | - | No significant change in BMI and BMI-Z between the IN groups compared to the CN groups |
| Novotny R / 2018 (19) | NR | - | No significant change in BMI-Z between the IN groups compared to the CN groups |
| Adab P /2018 (20) | NR | The duration of intervention was 12 months and fallowed after 15, 30 and 39 months from baseline | No significant change in BMI-Z between the IN groups compared to the CN groups |
| Sadeghi B / 2017 (21) | Low income | Analyzed separately in the girls and the boys | No significant change in BMI-Z between the IN groups compared to the CN groups |
| Swinburn B /2014 (22) | High income | - | No significant change in BMI and BMI-Z between the IN groups compared to the CN groups |
| Pettman T / 2014 (23) | NR | Was performed on preschool children (range of age: 4-5) and school children (range of age: 10-12) | No significant change in BMI and BMI-Z between the IN groups compared to the CN groups |
| Kremer P /2011 (24) | NR | - | No significant change in BMI and BMI-Z between the IN groups compared to the CN groups |
| Fotu K / 2011 (25) | NR | - | No significant change in BMI and BMI-Z between the IN groups compared to the CN groups |
| Sanigorski AM /2008 (26) | NR | - | ↓ BMI in the IN groups compared to the CN groups (p<0.05) |
| Romon M /2009 (27) | High income | The duration of intervention was 12 years and fallowed after 12 years from baseline (IAEI) and analyzed separately in the girls and the boys | ↓ BMI in boys and girls in the IN groups compared to the CN groups (p<0.05) |
| Crespo NC /2012(28) | NR | Was performed separately in community only (without educational session), family only and family community (in both educational session 22 hrs.) The duration of intervention was 1 years and fallowed after 1(IAEI),2 and 3 years from baseline. | No significant change in BMI-Z between the IN groups compared to the CN groups |
| Gentile DA / 2009 (29) | NR | The duration of intervention was 6 and 12 months and fallowed after 6 and 12 months from baseline. | No significant change in BMI between the IN groups compared to the CN groups |
| Economos CD /2007 (30) | NR | Was performed in two separate controls community, and analyzed separately in the girls and the boys | ↓ BMI-Z in boys and girls in the IN groups compared to the CN groups (p<0.05) |
| Wong WW /2016 (31) | Low income | The duration of intervention was 6 weeks (total increase of PA 18 and session 6 hrs.), 12 weeks (total Increase of PA 36 and educational session 12 hrs.), and 18 weeks (total increase of PA 54 and educational session 18 hrs.), and fallowed after 6, 12 and 18 weeks from baseline (IAEI). | No significant change in BMI and BMI-Z between the IN groups compared to the CN groups |
| Johnson BA / 2012 (32) | NR | - | ↓ BMI in the IN groups compared to the CN groups (p<0.05)  BMI was one of the outcoms, but its changes were not significantly investigated |
| de Silva-Sanigorski AM / 2010 (33) | Low income | Was performed on 2 years old and 3.5 years old children separately | ↓ BMI in the IN groups compared to the CN groups (only in 2 years old) (p<0.05)  ↓ BMI-Z in the IN groups compared to the CN groups (only in 2 years old) (p<0.05) |
| Taylor RW /2007 (34) | NR | The duration of intervention was 1 and 2 years and fallowed after 1 and 2 years from baseline (IAEI) | ↓ BMI in the IN groups compared to the CN groups (p<0.05) |
| de Henauw S /2015 (35) | NR | Analyzed separately in the girls and the boys | ↓ BMI-Z in boys and girls in the IN groups compared to the CN groups only in girls (p<0.05) |
| Elder JP /2014 (36) | NR | - | No significant change in BMI and BMI-Z between the IN groups compared to the CN groups |
| Eno Persson J /2018 (37) | NR | - | No significant change in BMI between the IN groups compared to the CN groups |
| Hammersley ML /2019 (38) | High income | The duration of intervention was 6 months and fallowed after 9 and 12 months from baseline. | No significant change in BMI between the IN groups compared to the CN groups |

| **Table S6.**  The general characteristics of the studies that not include in the meta-analysis | | | | | | | | | | | | | | | |
| --- | --- | --- | --- | --- | --- | --- | --- | --- | --- | --- | --- | --- | --- | --- | --- |
| **First Author /Year** | **Main focus** | **IN ^a^** | **Study type ^b^** | **Country /**  **Program name** | **Increase of PA ^c^** | **Session^d^** | **Sample size**  **(IN, CN)** | **Duration**  **(Year)** | **Range of age** | **Follow-up**  **(Year) ^e^** | **Frequency of Int. ^f^** | **Target group** | **Exclusion reason** | **^g^**  **BMI↓** | **^g^ BMI-Z**↓ |
| Chesham RA / 2018 (39) | PA | 2 | 1 | UK/  Daily Mile | 35 | NR | 379  (252,127) | 0.58 | 4-12 | 0 ^h^ | D | Children | Only report MD of BMI) not the values of BMI) | - | - |
| Waters E / 2017 (40) | Diet+PA | 2 | 1 | Australia /  Fun ‘n healthy in Moreland | NR | NR | 2965  (1426,  1539) | 5 | 5-12 | 0 | NR | Children | Only report MD of BMI and BMI-Z) not the values of them) | - | - |
| Lynch BA / 2016 (41) | Diet+PA | 0 | 1 | US/  Let's Go! 5-2-1-0 | NR | NR | 51  (29,22) | 0.33 | 7-8 | 0 | W | Children | Only report changes of BMI (not the values of BMI) | - | - |
| Lazorick S / 2016 (42) | Diet+PA | 1 | 1 | US/  MATCH | NR | NR | 362  (189,173) | 0.75 | 13-14 | 3.75 | W | Children | Only report changes of BMI and BMI-Z (not the values of BMI) | ✔ | ✔ |
| Santos RG / 2014 (43) | Diet+PA | 2 | 1 | Canada/  Healthy Buddies | 40 | 20,30 | 647  (340,307) | 0.83 | 6-8,9-12 | 0 | W | Children | Only report changes of BMI-Z (not the values of BMI-Z) | - | - |
| Rush E / 2014 (44) | Diet+PA | 2 | 1 | US/  Project Energize | NR | NR | 6629  (4804, 1825) | 2 | 6-8,9-11 | 5 | NR | Children | Only report MD of BMI) not the values of BMI) | - | - |
| Jordan KC / 2008 (45) | Diet+PA | 1 | 2 | US/  Gold Medal Schools Program | NR | NR | 411  (216,195) | 1 | 9-10 | 0 | NR | Children & Parents | Only report changes of BMI-Z (not the values of BMI-Z) | - | ✔ |
| Benjamin Neelon SE / 2015 (46) | PA | 3 | 1 | US/  Mebane on the Move | NR | NR | 104  (64,40) | 1 | 5-11 | 0 | D | Children | Only report MD of BMI-Z) not the values of BMI-Z) | - | ✔ |
| Karanja N / 2010 (47) | Diet | 3 | 1 | US/  TOTS | NR | NR | 410  (205,205) | 0.5 | 0-2 | 1.5 | D | Parents | Only report changes of BMI-Z (not the values of BMI-Z) | - | ✔ |
| Taveras EM /2017 (48) | Diet+PA | 3 | 2 | US/  MA-CORD | NR | NR | 6051  (1479, 4572) | 2 | 2-12 | 0 | D | Public health workers | Only report changes of BMI-Z (not the values of BMI-Z) | - | ✔ |
| Stookey JD / 2017 (49) | Diet+PA | 0 | 1 | US /  CCHP + HAP | NR | NR | 902  (522,380) | 3 | 2-5 | 0 | D | Public health workers | Only report changes of BMI-Z (not the values of BMI-Z) | - | ✔ |
| ^a^ : IN: intervention, 0: Only education,1: education as curricula, 2: education + change in school environment (such as increased PA or changes in school diet), 3: involvement other community sections).  ^b^ 1: Randomized controlled-trials (RCT), 2: Non-randomized controlled-trials  ^c^ Total hours increase of PA in the duration of intervention  ^d^ Session was held in the duration of intervention(hours)  ^e^ follow-up from end of intervention  ^f^ D: daily, W: weekly, M: monthly, NR: not reported.  ^g^ Tickets (**✔**) show a significant decrease (p< 0.05) in the body mass index (BMI) or BMI Z score (BMI-Z)  ^h^ Follow up 0 means: Immediately After the End of the Intervention | | | | | | | | | | | | | | | |

| **Table S7**. Publication bias checked by the Begg’s and Egger test in the BMI ^a^ and BMI-Z _score_ | | |
| --- | --- | --- |
| **Variables** | **Begg's P value** | **Egger P value** |
| **BMI** | 0.08 | 0.545 |
| **BMI-Z _score_** | 0.89 | 0.65 |
| ^a^ Body mass index | | |


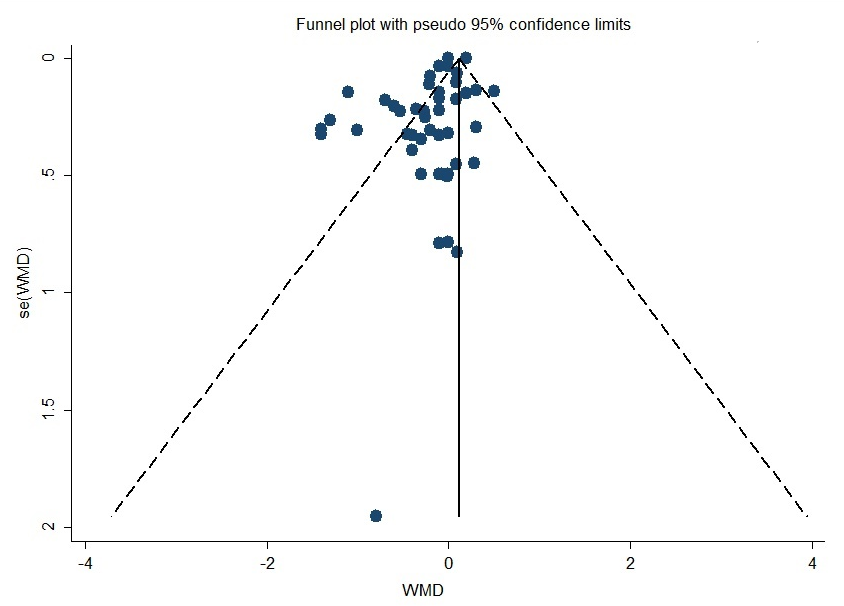


**A**


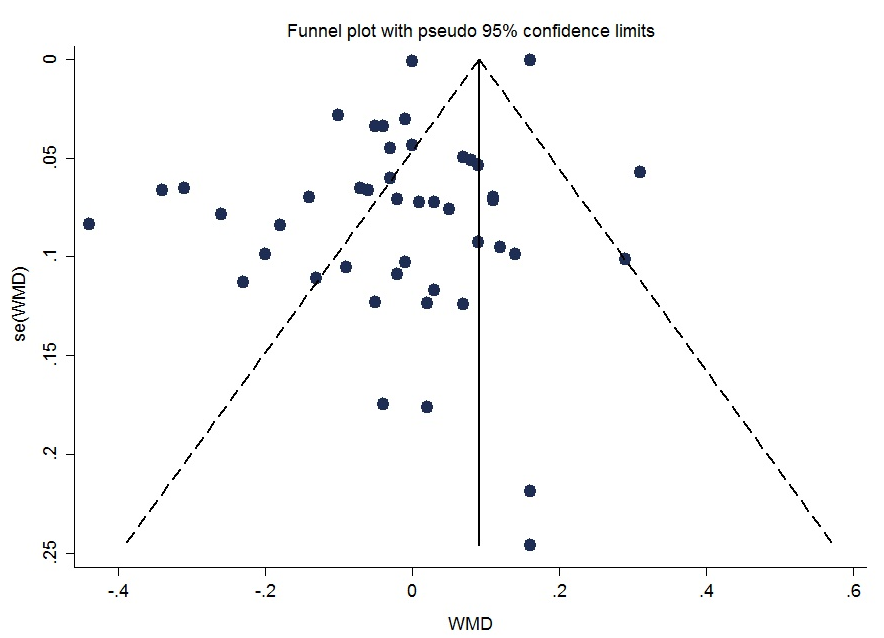


**B**

**Figure S1**. Begg's funnel plot (with pseudo 95% CIs) of the WMD versus the se (WMD) for studies evaluating the effects of obesity preventive policies in children and adolescents and (A) body mass index (BMI) (B) BMI-Z _score_

**Figure S2.** Sensitivity analysis for the effects of childhood obesity prevention policies on BMI-Z _score_

**References**

1. Wang Z, Xu F, Ye Q, Tse LA, Xue H, Tan Z, et al. Childhood obesity prevention through a community-based cluster randomized controlled physical activity intervention among schools in china: the health legacy project of the 2nd world summer youth olympic Games (YOG-Obesity study). Int J Obes. 2018;42(4):625-33.

2. Leme ACB, Baranowski T, Thompson D, Nicklas T, Philippi ST. Sustained impact of the “Healthy Habits, Healthy Girls – Brazil” school-based randomized controlled trial for adolescents living in low-income communities. Preventive Med Reports. 2018;10:346-52.

3. Lubans DR, Smith JJ, Plotnikoff RC, Dally KA, Okely AD, Salmon J, et al. Assessing the sustained impact of a school-based obesity prevention program for adolescent boys: The ATLAS cluster randomized controlled trial. Int J Behav Nutr Phys Act. 2016;13(1):92.

4. Hollis JL, Sutherland R, Campbell L, Morgan PJ, Lubans DR, Nathan N, et al. Effects of a 'school-based' physical activity intervention on adiposity in adolescents from economically disadvantaged communities: Secondary outcomes of the 'Physical Activity 4 Everyone' RCT. Int J Obes. 2016;40(10):1486-93.

5. Smith JJ, Morgan PJ, Plotnikoff RC, Dally KA, Salmon J, Okely AD, et al. Smart-phone obesity prevention trial for adolescent boys in low-income communities: The ATLAS RCT. Pediatrics. 2014;134(3):e723-e31.

6. Lubans DR, Morgan PJ, Okely AD, Dewar D, Collins CE, Batterham M, et al. Preventing obesity among adolescent girls: one-year outcomes of the nutrition and enjoyable activity for teen girls (NEAT Girls) cluster randomized controlled trial. Arch Pediatr Adolesc Med. 2012;166(9):821-7.

7. Millar L, Kremer P, de Silva-Sanigorski A, McCabe MP, Mavoa H, Moodie M, et al. Reduction in overweight and obesity from a 3-year community-based intervention in Australia: The 'It's Your Move!' project. Obes Rev. 2011;12(SUPPL. 2):20-8.

8. Llargues E, Franco R, Recasens A, Nadal A, Vila M, Pérez MJ, et al. Assessment of a school-based intervention in eating habits and physical activity in school children: The AVall study. J Epidemiol Community Health. 2011;65(10):896-901.

9. Salcedo Aguilar F, Martinez-Vizcaino V, Sanchez Lopez M, Solera Martinez M, Franquelo Gutierrez R, Serrano Martinez S, et al. Impact of an after-school physical activity program on obesity in children. The Journal of pediatrics. 2010;157(1):36-42.e3.

10. Neumark-Sztainer DR, Friend SE, Flattum CF, Hannan PJ, Story MT, Bauer KW, et al. New moves-preventing weight-related problems in adolescent girls: A group-randomized study. Am J Prev Med. 2010;39(5):421-32.

11. Group HS. A school-based intervention for diabetes risk reduction. N Engl J Med. 2010;363(5):443-53.

12. Dzewaltowski DA, Rosenkranz RR, Geller KS, Coleman KJ, Welk GJ, Hastmann TJ, et al. HOP'N after-school project: An obesity prevention randomized controlled trial. Int J Behav Nutr Phys Act. 2010;7(1):90.

13. Donnelly JE, Greene JL, Gibson CA, Smith BK, Washburn RA, Sullivan DK, et al. Physical Activity Across the Curriculum (PAAC): A randomized controlled trial to promote physical activity and diminish overweight and obesity in elementary school children. Prev Med: An International Journal Devoted to Practice and Theory. 2009;49(4):336-41.

14. Taylor R, McAuley K, Barbezat W, Farmer V, Williams S, Mann J. Two-year follow-up of an obesity prevention initiative in children: the APPLE project1-3. Am J Clin Nutr. 2008;88(5):1371-7.

15. Martínez Vizcaíno V, Salcedo Aguilar F, Franquelo Gutiérrez R, Solera Martínez M, Sánchez López M, Serrano Martínez S, et al. Assessment of an after-school physical activity program to prevent obesity among 9- to 10-year-old children: A cluster randomized trial. Int J Obes. 2008;32(1):12-22.

16. Foster GD, Sherman S, Borradaile KE, Grundy KM, Vander Veur SS, Nachmani J, et al. A policy-based school intervention to prevent overweight and obesity. Pediatrics. 2008;121(4):e794-e802.

17. Bell L, Ullah S, Leslie E, Magarey A, Olds T, Ratcliffe J, et al. Changes in weight status, quality of life and behaviours of South Australian primary school children: results from the Obesity Prevention and Lifestyle (OPAL) community intervention program. BMC Public Health. 2019;19(1):1338.

18. Santiago Felipe G, Rafael Casas E, Subirana I, Serra-Majem L, Torrent MF, Homs C, et al. Effect of a community-based childhood obesity intervention program on changes in anthropometric variables, incidence of obesity, and lifestyle choices in Spanish children aged 8 to 10&#xa0;years. Eur J Pediatr. 2018;177(10):1531-9.

19. Novotny R, Davis J, Butel J, Boushey CJ, Fialkowski MK, Nigg CR, et al. Effect of the Children's Healthy Living Program on Young Child Overweight, Obesity, and Acanthosis Nigricans in the US-Affiliated Pacific Region: A Randomized Clinical Trial. JAMA Netw Open. 2018;1(6):e183896.

20. Adab P, Pallan MJ, Lancashire ER, Hemming K, Frew E, Barrett T, et al. Effectiveness of a childhood obesity prevention programme delivered through schools, targeting 6 and 7 year olds: Cluster randomised controlled trial (WAVES study). BMJ (Online). 2018;360(8140):1-15.

21. Sadeghi B, Kaiser L, Schaefer S, Tseregounis I, Martinez L, Gomez‐Camacho R, et al. Multifaceted community‐based intervention reduces rate of BMI growth in obese Mexican‐origin boys. Pediatric obesity. 2017;12(3):247-56.

22. Swinburn B, Malakellis M, Moodie M, Waters E, Gibbs L, Millar L, et al. Large reductions in child overweight and obesity in intervention and comparison communities 3 years after a community project. Pediatric obesity. 2014;9(6):455-62.

23. Pettman T, Magarey A, Mastersson N, Wilson A, Dollman J. Improving weight status in childhood: Results from the eat well be active community programs. Int J Public Health. 2014;59(1):43-50.

24. Kremer P, Waqa G, Vanualailai N, Schultz JT, Roberts G, Moodie M, et al. Reducing unhealthy weight gain in Fijian adolescents: Results of the Healthy Youth Healthy Communities study. Obes Rev. 2011;12(SUPPL. 2):29-40.

25. Fotu K, Millar L, Mavoa H, Kremer P, Moodie M, Snowdon W, et al. Outcome results for the Ma'alahi Youth Project, a Tongan community‐based obesity prevention programme for adolescents. Obes Rev. 2011;12:41-50.

26. Sanigorski AM, Bell AC, Kremer PJ, Cuttler R, Swinburn BA. Reducing unhealthy weight gain in children through community capacity-building: Results of a quasi-experimental intervention program, Be Active Eat Well. Int J Obes. 2008;32(7):1060-7.

27. Romon M, Lommez A, Tafflet M, Basdevant A, Oppert JM, Bresson JL, et al. Downward trends in the prevalence of childhood overweight in the setting of 12-year school- and community-based programmes. Public Health Nutr. 2009;12(10):1735-42.

28. Crespo NC, Elder JP, Ayala GX, Slymen DJ, Campbell NR, Sallis JF, et al. Results of a multi-level intervention to prevent and control childhood obesity among Latino children: the Aventuras Para Niños Study. Ann Behav Med. 2012;43(1):84-100.

29. Gentile DA, Welk G, Eisenmann JC, Reimer RA, Walsh DA, Russell DW, et al. Evaluation of a multiple ecological level child obesity prevention program: Switch what you Do, View, and Chew. BMC medicine. 2009;7(1):49.

30. Economos CD, Hyatt RR, Goldberg JP, Must A, Naumova EN, Collins JJ, et al. A community intervention reduces BMI z-score in children: Shape up somerville first year results. Obesity. 2007;15(5):1325-36.

31. Wong WW, Ortiz CL, Stuff JE, Mikhail C, Lathan D, Moore LA, et al. A community-based healthy living promotion program improved self-esteem among minority children. J Pediatr Gastroenterol Nutr. 2016;63(1):106-12.

32. Johnson BA, Kremer PJ, Swinburn BA, De Silva-Sanigorski AM. Multilevel analysis of the Be Active Eat Well intervention: Environmental and behavioural influences on reductions in child obesity risk. Int J Obes. 2012;36(7):901-7.

33. de Silva-Sanigorski AM, Bell AC, Kremer P, Nichols M, Crellin M, Smith M, et al. Reducing obesity in early childhood: results from Romp & Chomp, an Australian community-wide intervention program. Am J Clin Nutr. 2010;91(4):831-40.

34. Taylor RW, McAuley KA, Barbezat W, Strong A, Williams SM, Mann JI. APPLE Project: 2-y findings of a community-based obesity prevention program in primary school-age children. Am J Clin Nutr. 2007;86(3):735-42.

35. de Henauw S, Huybrechts I, de Bourdeaudhuij I, Bammann K, Barba G, Lissner L, et al. Effects of a community-oriented obesity prevention programme on indicators of body fatness in preschool and primary school children. Main results from the IDEFICS study. Obes Rev. 2015;16:16-29.

36. Elder JP, Crespo NC, Corder K, Ayala GX, Slymen DJ, Lopez NV, et al. Childhood obesity prevention and control in city recreation centres and family homes: The MOVE/me Muevo Project. Pediatric obesity. 2014;9(3):218-31.

37. Eno Persson J, Bohman B, Tynelius P, Rasmussen F, Ghaderi A. Prevention of Childhood Obesity in Child Health Services: Follow-Up of the PRIMROSE Trial. Childhood obesity (Print). 2018;14(2):99-105.

38. Hammersley ML, Okely AD, Batterham MJ, Jones RA. An internet-based childhood obesity prevention program (TIMe2bhealthy) for parents of preschool-aged children: Randomized controlled trial. J Med Internet Res. 2019;21(2):e11964.

39. Chesham RA, Booth JN, Sweeney EL, Ryde GC, Gorely T, Brooks NE, et al. The Daily Mile makes primary school children more active, less sedentary and improves their fitness and body composition: A quasi-experimental pilot study. BMC Med. 2018;16(1).

40. Waters E, Gibbs L, Tadic M, Ukoumunne OC, Magarey A, Okely AD, et al. Cluster randomised trial of a school-community child health promotion and obesity prevention intervention: findings from the evaluation of fun ‘n healthy in Moreland! BMC Public Health. 2018;18(1):92.

41. Lynch BA, Gentile N, Maxson J, Quigg S, Swenson L, Kaufman T. Elementary School-Based Obesity Intervention Using an Educational Curriculum. Journal of primary care & community health. 2016;7(4):265-71.

42. Lazorick S, Fang X, Crawford Y. The MATCH Program: Long-Term Obesity Prevention Through a Middle School Based Intervention. Childhood obesity (Print). 2016;12(2):103-12.

43. Santos RG, Durksen A, Rabbanni R, Chanoine JP, Miln AL, Mayer T, et al. Effectiveness of peer-based healthy living lesson plans on anthropometric measures and physical activity in elementary school students a cluster randomized trial. JAMA Pediatrics. 2014;168(4):330-7.

44. Rush E, McLennan S, Obolonkin V, Vandal AC, Hamlin M, Simmons D, et al. Project Energize: whole-region primary school nutrition and physical activity programme; evaluation of body size and fitness 5 years after the randomised controlled trial. Br J Nutr. 2014;111(2):363-71.

45. Jordan KC, Erickson ED, Cox R, Carlson EC, Heap E, Friedrichs M, et al. Evaluation of the gold medal schools program. Journal of the American Dietetic Association. 2008;108(11):1916-20.

46. Benjamin Neelon SE, Namenek Brouwer RJ, Østbye T, Evenson KR, Neelon B, Martinie A, et al. A community-based intervention increases physical activity and reduces obesity in school-age children in North Carolina. Childhood Obesity. 2015;11(3):297-303.

47. Karanja N, Lutz T, Ritenbaugh C, Maupome G, Jones J, Becker T, et al. The TOTS community intervention to prevent overweight in American Indian toddlers beginning at birth: a feasibility and efficacy study. Journal of community health. 2010;35(6):667-75.

48. Taveras EM, Perkins M, Anand S, Woo Baidal JA, Nelson CC, Kamdar N, et al. Clinical effectiveness of the Massachusetts Childhood Obesity Research Demonstration initiative among low‐income children. Obesity. 2017;25(7):1159-66.

49. Stookey JD, Evans J, Chan C, Tao-Lew L, Arana T, Arthur S. Healthy apple program to support child care centers to alter nutrition and physical activity practices and improve child weight: A cluster randomized trial. BMC Public Health. 2017;17(1):965.
